# Eomes cannot replace its paralog T-bet during expansion and differentiation of CD8 effector T cells

**DOI:** 10.1371/journal.ppat.1008870

**Published:** 2020-09-29

**Authors:** Jonas Fixemer, Jonas F. Hummel, Frederic Arnold, Christoph S. N. Klose, Alexis Hofherr, Kristoffer Weissert, Tamara Kögl, Michael Köttgen, Sebastian J. Arnold, Peter Aichele, Yakup Tanriver

**Affiliations:** 1 Institute of Medical Microbiology and Hygiene, Faculty of Medicine, University of Freiburg, Freiburg, Germany; 2 Department of Internal Medicine IV, Medical Center—University of Freiburg, Faculty of Medicine, University of Freiburg, Freiburg, Germany; 3 Berta-Ottenstein-Programme, Faculty of Medicine, University of Freiburg, Freiburg, Germany; 4 Institute of Microbiology, Infectious Diseases and Immunology, Charité - Universitätsmedizin Berlin, Berlin, Germany; 5 Institute for Immunodeficiency, Center for Chronic Immunodeficiency (CCI), Medical Center—University of Freiburg, Faculty of Medicine, University of Freiburg, Freiburg, Germany; 6 Faculty of Biology, University of Freiburg, Freiburg, Germany; 7 CIBSS—Centre for Integrative Biological Signalling Studies, University of Freiburg, Freiburg, Germany; 8 Institute of Experimental and Clinical Pharmacology and Toxicology, Faculty of Medicine, University of Freiburg, Freiburg, Germany; ETH Zurich, SWITZERLAND

## Abstract

The two T-box transcription factors T-bet and Eomesodermin (Eomes) are important regulators of cytotoxic lymphocytes (CTLs), such as activated CD8 T cells, which are essential in the fight against intracellular pathogens and tumors. Both transcription factors share a great degree of homology based on sequence analysis and as a result exert partial functional redundancy during viral infection. However, the actual degree of redundancy between T-bet and Eomes remains a matter of debate and is further confounded by their distinct spatiotemporal expression pattern in activated CD8 T cells. To directly investigate the functional overlap of these transcription factors, we generated a new mouse model in which *Eomes* expression is under the transcriptional control of the endogenous *Tbx21* (encoding for T-bet) locus. Applying this model, we demonstrate that the induction of Eomes *in lieu* of T-bet cannot rescue T-bet deficiency in CD8 T cells during acute lymphocytic choriomeningitis virus (LCMV) infection. We found that the expression of Eomes instead of T-bet was not sufficient for early cell expansion or effector cell differentiation. Finally, we show that imposed expression of Eomes after acute viral infection promotes some features of exhaustion but must act in concert with other factors during chronic viral infection to establish all hallmarks of exhaustion. In summary, our results clearly underline the importance of T-bet in guiding canonical CTL development during acute viral infections.

## Author summary

According to the World Health Organization infectious diseases kill over 17 million people per year. At the same time highly infectious viral diseases, such as Ebola and COVID-19 that are lacking specific treatments, are emerging to pose additional threats. It is therefore pivotal to precisely understand how our immune system responds towards pathogens to develop new treatment options. Here we have investigated the role of two related molecules, named T-bet and Eomes, that guide the development and function of lymphocytes in their fight against intracellular pathogens. We specifically focused on cytotoxic lymphocytes as these cells dominate the early phase of viral containment. We show that T-bet is essential for the expansion of cytotoxic lymphocytes and equip lymphocytes with the ability to efficiently eliminate virus-infected cells. Hence, our study provides new insights into the importance and specific actions of T-bet during acute viral infections and how this might be harnessed for future therapeutic interventions.

## Introduction

The transcription factors (TFs) T-bet and Eomesodermin (Eomes) regulate type 1 immune responses in innate [1] and adaptive lymphocytes [2]. Such responses are broadly characterized by the signature cytokine IFN-γ and cytotoxic effector function against intracellular pathogens [3]. T-bet and Eomes both belong to the large and diverse T-box TF family, that all share the evolutionary conserved DNA-binding domain, the T-box. T-box TFs are present in all metazoans and play important roles in cell fate decisions and organogenesis during development. T-box genes can be grouped into five subfamilies and genes within the same subfamily have arisen from duplication of a single ancestral gene [4]. T-bet and Eomes together with Tbr1 belong to the Tbr1 subfamily and are thus paralogs that share a high degree of sequence homology, especially within their T-box domains. Nevertheless, their regulation and expression within the immune system is quite distinct, which implies unique functions [5–7]. T-bet was initially identified as the lineage specifying TF of Th1 CD4 T cells [2], who could also repress alternative cell fates like Th2 or Th17. However, T-bet’s role extends beyond CD4 T cells as it is also expressed in activated CD8 T cells [8], innate-like T cells (e.g. NKT cells) [9], CD8αα T cells [10], B cells [11], innate lymphoid cells (ILCs) [1], and even non-lymphoid cells like dendritic cells [12]. Accordingly, T-bet deficiency has broad implications for infectious diseases, immunopathology and anti-tumor immunity. In contrast, Eomes has a much more restricted expression pattern. Although partly expressed in certain CD4 [13, 14] and γδ T cells subsets [15], Eomes is largely confined to conventional NK (cNK) cells [5] and activated or memory CD8 T cells [16], both of which also express T-bet. Based on experiments with the respective knockout mice, it has been argued that T-bet and Eomes have partially redundant and overlapping functions in CD8 T cells and can therefore compensate for each other [3]. Initially, this has been investigated during acute viral infections with lymphocytic choriomeningitis virus (LCMV) Armstrong (Arm). This infection leads to the differentiation of naïve CD8 T cells into cytotoxic T cells (CTLs), whose two cardinal features, cytotoxicity and cytokine production, are controlled by T-box TF and are essential for viral control [7]. Interestingly, mice that lack either T-bet or Eomes in their T cell compartment show apparently no defect in the early production of IFN-γ or cytotoxic molecules and, similar to wild type mice, show no viral replication in spleen and kidney after three weeks [3]. Yet, if mice lack both, T-bet and Eomes, they fail to produce sufficient amounts of IFN-γ or to execute sufficient cytotoxicity and are therefore unable to control viral replication. Moreover, activated T cells from these mice show an aberrant type 17 response that leads to progressive multi-organ infiltration with neutrophils and early death [3]. It was therefore concluded that T-bet and Eomes both can regulate early differentiation of CTLs and are therefore redundant during acute infection with intracellular pathogens. However, later it became clear that these two TFs are not simultaneously and uniformly expressed in activated CD8 T cells. Whereas virtually all activated CD8 T cells express T-bet to different degrees, only a subpopulation additionally expresses Eomes [7]. This heterogeneity might be partially explained by increased T cell receptor signaling [17], abundance of inflammatory cytokines, like IL-12 [18], or heightened mTOR kinase activity [19], which favor the induction of T-bet in CTLs while repressing Eomes.

Although numerous models of early T cell differentiation have been developed, T-bet is well known for its role in the formation of short-lived effector cells (SLECs), which are KLRG1^+^ CD127^-^ and do not develop in *Tbx21*^*-/-*^ (*Tbx21* encodes for T-bet) mice [20]. This phenotype already indicates that T-bet is essential during acute infection with LCMV Arm and that Eomes only provides partial redundancy here. In contrast, memory precursor effector cells (MPECs), which are KLRG1^-^ CD127^+^, and early effector cells (EECs), which are KLRG1^-^ CD127^-^ are not reduced in *Tbx21*^*-/-*^ mice. Intriguingly, Eomes is equally expressed in a subpopulation of SLECs and MPECs and T cell specific Eomes deficient mice show no perturbations in SLEC and MPEC formation during acute infection [7]. However, Eomes becomes vital after viral clearance, after which the percentage of Eomes expressing CD8 T cells increases with time after infection. This process intrinsically requires Eomes, as Eomes deficient memory T cells show defects in long-term maintenance and re-expansion upon secondary challenge when competing with wild type T cells [21].

These conclusions were further corroborated by lineage-tracing experiments in Eomes deficient mice at steady-state. It has been shown that CD44^+^ CD62L^+^ central memory CD8 T cells (T_CM_) show the highest percentage of Eomes expressing cells; followed by CD44^+^ CD62L^-^ effector memory CD8 T cells (T_EM_), whereas CD44^-^ CD62L^+^ naïve CD8 T cells do not express Eomes. Intriguingly, it was demonstrated that Eomes was not a mere marker of cells when expressed, but that Eomes^+^ memory T cells at steady state, similar to Eomes^+^ memory T cells after an acute infection, depend on Eomes for their survival or development [22].

Further insights into specific functions of T-bet and Eomes came from mice, which were chronically infected with LCMV clone 13 (cl13). The partially inverse expression pattern of T-bet and Eomes can be used to identify two distinct transcriptional states: T-bet^high^ Eomes^low^ and T-bet^low^ Eomes^high^ memory CD8 T cells that cooperatively sustain an effective antiviral immune response [22]. Moreover, using additional markers to discern T-bet^low^ Eomes^high^ CD8 memory T cells it has been demonstrated that Eomes is not only essential for the development of this cellular subset but also for its maintenance, once it is established [23]. In mice chronically infected with LCMV cl13, T-bet^low^ Eomes^high^ cells accumulate with time after infection and show characteristics of exhaustion, such as high expression of PD-1, LAG-3, TIM3, TIGIT, CD39, CD160, CTLA-4, as well as reduced production of pro-inflammatory cytokines and only moderate proliferation potential as compared to T-bet^high^ Eomes^low^ CD8 memory T cells [7, 23]. Apparently, this phenotype allows for infection control without causing overt immunopathology during progressive disease.

In summary, previous studies suggested that T-bet preferentially drives a program of terminal effector differentiation while Eomes supports a program of long-term maintenance with adapted effector function. However, it has to be emphasized that activated T-bet^high^ Eomes^low^ and T-bet^low^ Eomes^high^ CD8 T cells engage distinct transcriptional networks that involve various additional transcriptional regulators [24], e.g. Id3, Bcl6, Tcf1, Blimp-1, IRF4, TOX, which contribute to transcriptional and functional differences between these populations. Nonetheless, this plethora of observations clearly indicates that despite their sequence homology and partial redundancy T-bet and Eomes delineate two different effector cell populations. As described above, a key challenge of interpreting previous results with respect to the functional redundancy of T-bet during acute infection is the heterogeneous expression pattern of Eomes in activated CD8 T cells. *Bona fide* functional redundancy on a transcriptional level would predict that when an activated CD8 T cell cannot express T-bet, e.g. *Tbx21*^*-/-*^ CD8 T cell, it will by default express Eomes, which then coordinates a transcriptional and, eventually, functional program similar to T-bet. Yet, it is not fully clear whether activated CD8 T cells in *Tbx21*^*-/-*^ mice uniformly express Eomes. This alternative expression pattern would be an important pre-condition to directly test and compare the transcriptional activities of T-bet and Eomes at the single cell level. However, since no such studies have been performed so far *in vitro* or *in vivo*, the degree of functional redundancy of T-box TFs within the immune system remains a matter of debate.

To address the redundancy of T-bet and Eomes, we generated a novel model, in which the *Eomes* coding sequence was introduced into the first exon of the *Tbx21* locus followed by a P2A sequence and *mCherry* as fluorescent reporter (*Tbx21*^*Eomes-P2A-mCherry/+*^, hereafter called *Tbx21*^*E*^). This allele results in a null allele for T-bet and the synchronized expression of Eomes *in lieu* of T-bet as the *Tbx21*^*E*^ allele is entirely under the transcriptional control of the *Tbx21* locus. We then went on to stringently test the functional redundancy of T-bet during acute infection with LCMV WE, by comparing *Tbx21*^*+/+*^ wild type with *Tbx21*^*-/-*^ and *Tbx21*^*E/E*^ mice. Finally, to exclude extrinsic effects of our *Tbx21*^*E*^ allele or the state of infection on activated CD8 T cells, we investigated the redundancy of T-bet in an adoptive transfer model.

The results presented here unequivocally show that Eomes cannot properly compensate for the loss of T-bet in activated CD8 T cells during acute infection with LCMV WE but rather promotes an exhaustion-like phenotype. In summary, this study underlines the importance of the diversification of the Tbr1 T-box TF family in the immune system at the onset of vertebrate evolution [25].

## Results

### Generation of a novel mouse model by putting *Eomes* expression under control of the endogenous *Tbx21* locus

**Fig 1:**
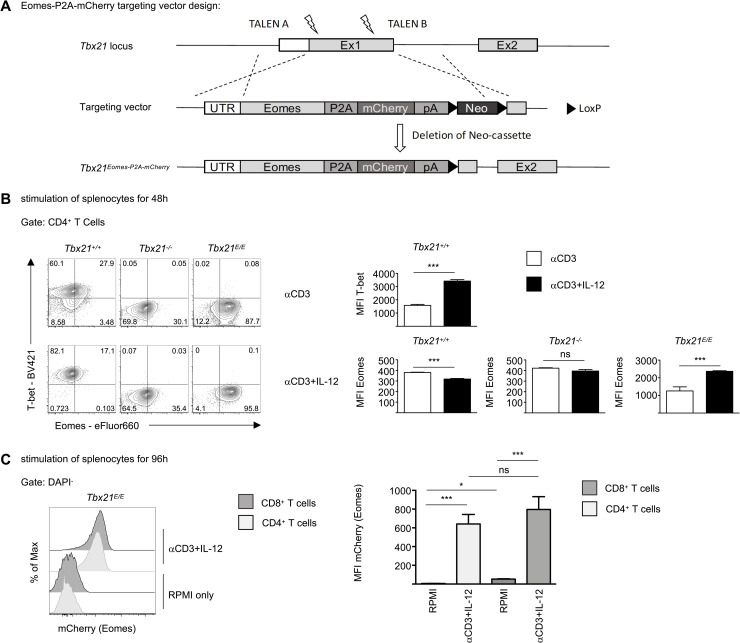
Generation and validation of novel *Tbx21*^*Eomes-P2A-mCherry/+*^ mouse. (A) Schematic design of the *Eomes-P2A-mCherry* target vector and insertion of the *Eomes-P2A-mCherry* allele into the *Tbx21* locus of ES cells by TALEN-induced recombinase. The deletion of the neomycin cassette was achieved by crossing mice with a Sox2-Cre mouse line. (B) Splenocytes of indicated groups were stimulated *in vitro* with soluble anti-CD3 with or without IL-12. After 48 hours expression of T-bet and Eomes of CD4^+^ T cells was analyzed by flow cytometry after intranuclear staining as depicted in contour plots. Bar diagrams show mean fluorescence intensities (MFIs) of T-bet (top row) or MFI of Eomes (bottom row) of CD4^+^ T cells of indicated groups. Gating for Eomes was set according to Eomes-deficient splenocytes (*T-cre Eomes*^*flox/flox*^). (C) Splenocytes of *Tbx21*^*E/E*^ mice were cultured *in vitro* with soluble anti-CD3 and IL-12 or RPMI only for 96 hours. Histogram shows expression of mCherry of indicated DAPI^-^ T cell subsets as determined by flow cytometry. Bar diagram shows MFI of mCherry of indicated T cell subsets. Statistical analysis: *p < 0.05; **p < 0.01; ***p < 0.001; ns, not significant; two-tailed unpaired Student`s *t*-test; Error bars denote mean + SEM. (B, C) Data are representative of 3 independent experiments with in total 8–9 mice per group.

Previously, a *Tbx21-Eomes* transgenic mouse model had been generated by using a bacterial artificial chromosome (BAC) approach, in which expression of *Eomes* was put under the transcriptional control of the *Tbx21* regulatory elements of the BAC transgene [26]. However, such an approach does not tightly control for copy numbers and insertion, which might produce several off-target effects. Moreover, this strategy does not distinguish between transgenic and endogenous Eomes expression. To study the functional redundancy of T-bet in activated CD8 T cells, we therefore used TALEN-guided knock-in technology [27] to insert the *Eomes* coding sequence into the *Tbx21* locus followed by a *P2*A peptide-sequence and the *mCherry* reporter to generate the *Tbx21*^*Eomes-P2A-mCherry/+*^ allele (hereafter called *Tbx21*^*E*^; Fig 1A). *Tbx21*^*E/+*^ heterozygous mice were interbred to obtain *Tbx21*^*E/E*^ mice, that homozygously express *Eomes* from the *Tbx21* locus. Hence, these mice were also deficient for *Tbx21*. Mice were born at mendelian ratio and showed no developmental abnormalities or clinical immunopathology during a 3-year observation period. The *Tbx21*^*E*^ allele had no effect on absolute cell numbers in spleen, lymph nodes or thymus (S1A Fig). Furthermore, the thymic distribution of CD4^+^ single positive (SP), CD8^+^ SP, CD4^+^ CD8^+^ double positive and CD4^-^ CD8^-^ double negative cells was unperturbed (S1B Fig). Similarly, proportions of CD4 and CD8 T cells as well as B cells in spleen (S1C Fig) and lymph nodes (S1D Fig) showed only minor differences. The *Tbx21*^*E*^ allele was associated with more T_CM_ (CD44^+^ CD62L^+^), which was most pronounced in *Tbx21*^*E/E*^ mice and in line with the notion that Eomes promotes maintenance of T_CM_ in wild type mice. Finally, expression of the *Tbx21*^*E*^ allele had no effect on the frequency of CD4^+^ CD25^+^ regulatory T cells in spleen and lymph nodes. In summary, Eomes expression from the *Tbx21*^*E*^ allele showed no global effects on the adaptive immune system at steady state. Detailed characterization of innate and innate-like lymphocytes in these mouse strains will be reported elsewhere. To test, whether the *Tbx21*^*E*^ allele was truly under the transcriptional control of the *Tbx21* locus, we stimulated splenocytes from *Tb21*^*+/+*^, *Tbx21*^*-/-*^ and *Tbx21*^*E/E*^ mice with anti-CD3 with or without IL-12. IL-12 signals via STAT4 and it has been shown for CD4 [28] and CD8 T cells [20] that it is a strong inductor of T-bet. Additionally, IL-12 represses Eomes in CD8 T cells [18]. Stimulation with anti-CD3 alone led to uniform expression of T-bet in CD4 T cells from *Tbx21*^*+/+*^ mice (Fig 1B), which was further increased by IL-12 (Fig 1B). As control *Tbx21*^*-/-*^ and *Tbx21*^*E/E*^ showed no expression of T-bet in any condition. Moreover, stimulation of *Tbx21*^*+/+*^ cells induced low levels of Eomes in CD4 T cells, which was repressed by additional IL-12 (Fig 1B). Intriguingly, stimulation of *Tbx21*^*-/-*^ cells with anti-CD3 did not lead to an increase of Eomes expression, arguing against a default expression of Eomes in the absence of T-bet (Fig 1B). As predicted, stimulated CD4 T cells from *Tbx21*^*E/E*^ mice showed uniform and high expression of Eomes, which was further boosted by IL-12 (Fig 1B). Hence, Eomes expression from the *Tbx21*^*E*^ locus recapitulates the expression pattern of T-bet expression in *Tbx21*^*+/+*^ CD4 T cells.

Next, we explored the faithfulness of the mCherry reporter in CD8 and cNK cells, as these cells typically express T-bet. At steady state T-bet showed the highest expression in cNK cells, intermediate levels in CD8 memory and was not detectable in naïve wild type CD8 T cells. Correspondingly, mCherry showed the strongest signal in cNK cells and no signal in naïve CD8 T cells (S1E and S1F Fig). Memory CD8 T cells at steady-state showed an intermediate signal-strength. However, this expression could be boosted by polyclonal stimulation with anti-CD3 in combination with IL-12 (Fig 1C). Similar results were obtained with CD4 T cells (Fig 1C). In summary we can conclude, that the expression of Eomes and mCherry under the transcriptional control of the *Tbx21* locus is reflecting T-bet expression and is dynamically regulated in response to T cell receptor (TCR) stimulation and cytokine signaling.

### T-bet deficient *Tbx21*^*E/E*^ mice fail to rescue the expansion and differentiation deficit of *Tbx21*^*-/-*^ mice

**Fig 2:**
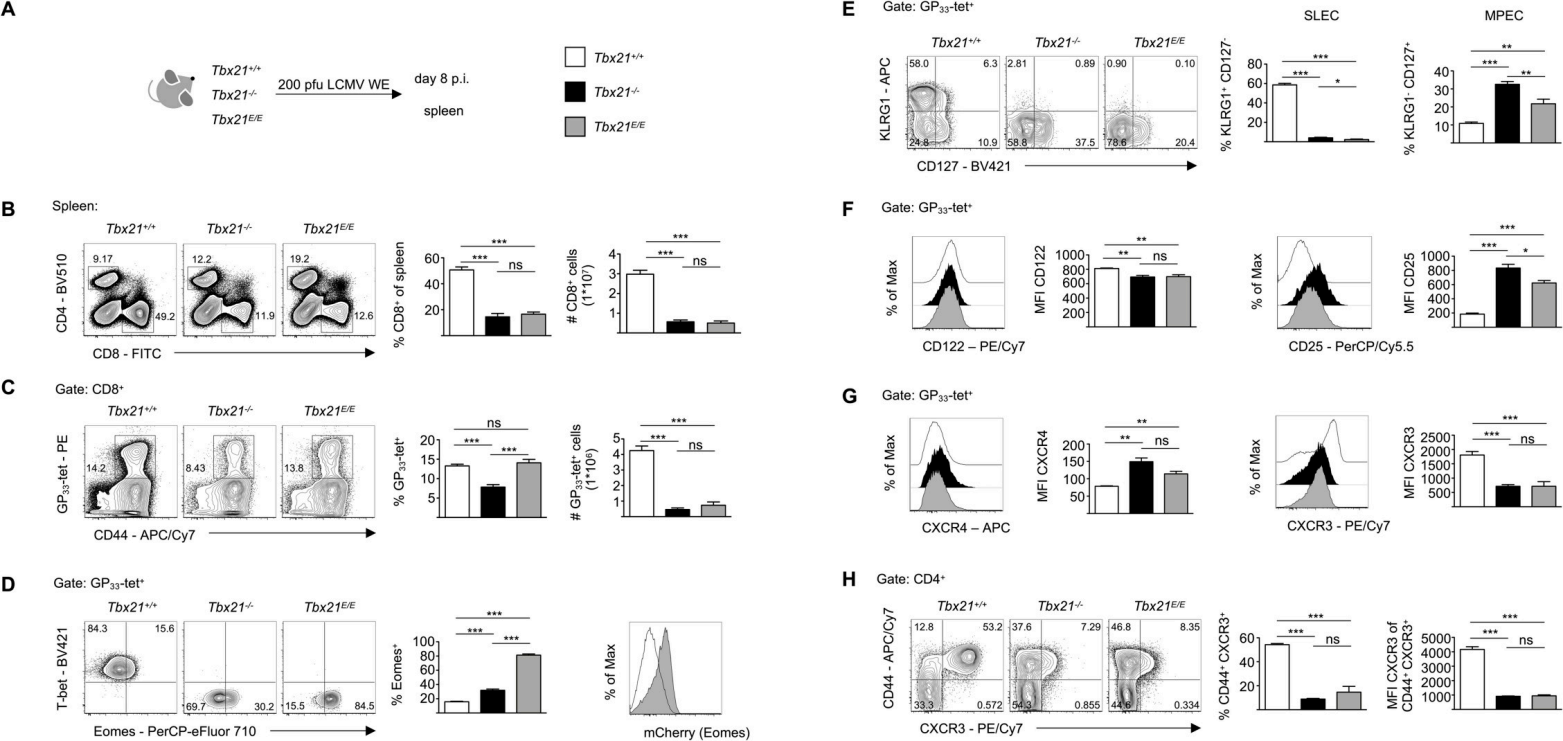
Expansion and differentiation of SLECs is dependent on T-bet. (A) Age- and sex-matched *Tbx21*^*+/+*^, *Tbx21*^*-/-*^ and *Tbx21*^*E/E*^ mice were infected with 200 pfu LCMV WE. At day 8 p.i. splenocytes were analyzed by flow cytometry. (B) Contour plots show frequency of CD4^+^ and CD8^+^ T cells. Bar diagrams show frequency and absolute number (#) of CD8^+^ T cells. (C) Contour plots show frequency of GP_33_-tetramer^+^ CD44^+^ (GP_33_-tet^+^) CD8^+^ T cells. Bar diagrams show frequency and absolute number (#) of indicated cells. (D) Contour plots show expression of T-bet and Eomes of GP_33_-tet^+^ CD8^+^ T cells as determined by flow cytometry after intranuclear staining. Quadrant gates were set according to CD44^-^ CD4^+^ T cells (T-bet^-^ Eomes^-^ cells). Bar diagram shows percentage of Eomes^+^ cells. Histogram shows MFI of mCherry expression. (E) Contour plots show expression of KLRG1 and CD127 of GP_33_-tet^+^ CD8^+^ T cells. Bar diagrams show percentage of SLEC (KLRG1^+^ CD127^-^) and MPEC (KLRG1^-^ CD127^+^) subsets. (F) Histograms show expression of CD122 and CD25 of GP_33_-tet^+^ CD8^+^ T cells and quantification of MFI of CD122 and CD25 via bar diagrams. (G) Histograms show expression of CXCR4 and CXCR3 of GP_33_-tet^+^ CD8^+^ T cells and MFI of CXCR4 and CXCR3 of GP_33_-tet^+^ CD8^+^ T cells depicted as bar diagrams. (H) Contour plots show expression of CD44 and CXCR3 of CD4^+^ T cells. Bar diagrams show frequency of CD44^+^ CXCR3^+^ CD4^+^ T cells and MFI of CXCR3 of CD44^+^ CXCR3^+^ CD4^+^ T cells. Statistical analysis: *p < 0.05; **p < 0.01; ***p < 0.001; ns, not significant; two-tailed unpaired Student`s *t*-test; Error bars denote mean + SEM. (B-H) Data are representative of 3 independent experiments with in total 8–9 mice per group.

T-bet plays an important role during the acute phase of an infection by guiding the development of CTLs [8]. We therefore investigated its redundancy in LCMV WE low dose infection, which is usually controlled within 8 days after infection in *Tbx21*^*+/+*^ wild type mice. Hence, we infected *Tbx21*^*+/+*^, *Tbx21*^*-/-*^ and *Tbx21*^*E/E*^ mice with 200 plaque forming units (pfu) LCMV WE and analyzed infected mice 8 days later (Fig 2A). Upon gross examination it was apparent, that *Tbx21*^*+/+*^ wild type mice developed a massive splenomegaly, which was strongly curtailed in either *Tbx21* deficient strain (*Tbx21*^*-/-*^ and *Tbx21*^*E/E*^) (S2A Fig). This was partially the result of a failed CD8 T cell expansion, with *Tbx21*^*+/+*^ wild type mice having around 5-times more CD8 T cells than *Tbx21*^*-/-*^ or *Tbx21*^*E/E*^ mice (Fig 2B), whereas the absolute numbers of CD4 T cells was not different among the three strains (S2A Fig). Correspondingly, we detected a massive reduction of LCMV glycoprotein 33–41 epitope (GP_33_)-specific CD8 T cells (GP_33_-tet^+^) in *Tbx21*^*-/-*^ and *Tbx21*^*E/E*^ mice (Fig 2C). Despite the striking difference in absolute numbers the frequencies of GP_33_-tet^+^ CD8 T cells were similar in *Tbx21*^*+/+*^ and *Tbx21*^*E/E*^ mice (Fig 2C) and significantly higher than in *Tbx21*^*-/-*^ mice. Intranuclear staining for T-bet and Eomes in GP_33_-tet^+^ CD8 T cells from *Tbx21*^*+/+*^ wild type mice at day 8 revealed, that they uniformly expressed T-bet, but only a subpopulation was additionally positive for Eomes (Fig 2D). The latter was also confirmed in *Eomes*^*Gfp/+*^ reporter mice [29], which demonstrated that at day 8 of an acute infection around 15% of GP_33_-tet^+^ CD8 T cells are T-bet^+^ Eomes^+^ (GFP^+^), with the remaining cells being T-bet^+^ Eomes^-^ (GFP^-^) (S2B Fig). GP_33_-tet^+^ CD8 T cells in *Tbx21*^*-/-*^ mice showed a significant increase in the percentage of Eomes^+^ cells, however, the majority of cells (~70%) remained Eomes^-^ (Fig 2D). Hence, we can conclude that the absence of T-bet does not result in the mandatory expression of Eomes and therefore Eomes, by definition, cannot compensate for the actions of T-bet in the majority of CD8 T cells during an acute infection with LCMV WE.

As expected, GP_33_-tet^+^ CD8 T cells from *Tbx21*^*E/E*^ mice showed a uniform expression of Eomes and mCherry, but no expression of T-bet (Fig 2D). A similar expression pattern of Eomes was also seen in activated GP_33_-tet^-^ CD8 T cells from the three genotypes (S2C Fig). However, this expression of Eomes was not sufficient to rescue the development of SLECs, which were also absent in *Tbx21*^*E/E*^ mice (Fig 2E). Intriguingly, *Tbx21*^*E/E*^ mice had a lower frequency of MPECs than *Tbx21*^*-/-*^ mice, but still more than *Tbx21*^*+/+*^ wild type mice (Fig 2E). T-bet and Eomes guide the differentiation and migration of T cells by regulating their responsiveness towards cytokines and chemokines, respectively. The expression of CD122 (IL-2Rβ chain), which is essential for IL-2 and IL-15 signaling, can be cooperatively regulated by the two TFs [16], however, during acute infection T-bet deficiency resulted in a significant reduction of CD122 expression, which could not be rescued by Eomes in *Tbx21*^*E/E*^ mice (Fig 2F, left). The reduced expression of CD122 in T-bet deficient strains could have been due to the absence of SLECs in these strains. However, co-staining of CD127 and CD122 in GP_33_-tet^+^ CTLs clearly showed that MPECs (CD127^+^) had the lowest expression of CD122 in T-bet deficient strains, whereas there was no difference in the CD122 expression of CD127^-^ CD8 T cells, including SLECs and EECs (S2D Fig). Hence, T-bet not only regulates SLEC formation but is also relevant for appropriate CD122 expression in MPECs. In contrast, CD25 (IL-2Rα chain), which is essential for IL-2 signaling, was upregulated in *Tbx21*^*-/-*^ mice, which was partially curtailed in *Tbx21*^*E/E*^ mice (Fig 2F, right). The chemokine receptor CXCR4 controls homing of T cells, especially to the bone marrow, where one of its ligands, CXCL12, is highly expressed. Published reports have indicated that CXCR4 expression in immune [30] and non-immune [31] cells can be positively regulated by Eomes. However, this is not an absolute requirement, as even Eomes^-^ naïve T cells or thymocytes express relevant levels of CXCR4 [32]. CXCR4 was downregulated in GP_33_-tet^+^ CD8 T cells from *Tbx21*^*+/+*^ wild type mice, but remained elevated in CD8 T cells from *Tbx21*^*-/-*^ and *Tbx21*^*E/E*^ mice (Fig 2G, left). Interestingly, there was no further increase in *Tbx21*^*E/E*^ mice compared to *Tbx21*^*-/-*^ mice. Th1 cells express the chemokine receptor CXCR3, which guides them towards sites of inflammation [33]. High expression was also completely dependent on T-bet and was therefore lost in GP_33_-tet^+^ CD8 T cells in *Tbx21*^*-/-*^ and *Tbx21*^*E/E*^ mice (Fig 2G, right). Similar results were also observed in activated polyclonal CD44^+^ CD4 T cells from the three strains (Fig 2H). In summary, we show here that despite the strong and uniform induction of Eomes in CD8 T cells from *Tbx21*^*E/E*^ mice, we did not observe a rescue of the *Tbx21*^*-/-*^ phenotype in the context of LCMV infection with respect to clonal expansion, effector differentiation and responsiveness towards cytokines or chemokines. This would be in line with a model, in which there is a only limited functional overlap in the transcriptional activity of T-bet and Eomes [34].

### T-bet deficient strains show signs of T cells exhaustion and fail to clear acute LCMV WE infection

**Fig 3:**
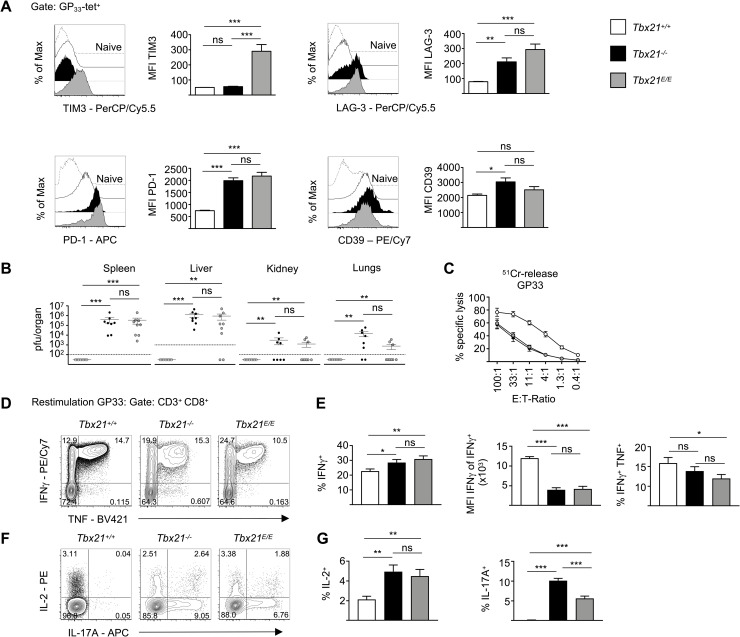
T-bet is essential for viral clearance of acute LCMV infection. (A) *Tbx21*^*+/+*^, *Tbx21*^*-/-*^ and *Tbx21*^*E/E*^ mice were infected as in Fig 2. Histograms show expression of indicated receptor of GP_33_-tet^+^ CD8 T cells. Dashed lines serve as control and refer to naïve (CD44^-^) CD8 T cells. (B) Viral titers in indicated organs determined by standard focus-forming assay. Error bars represent mean ± SEM. Symbols represent the values of individual mice. Dashed lines represent detection limit. (C) Cytolytic activity of CD8 T cells was determined on GP33-peptide loaded EL-4 target cells in a 5-hour-^51^Cr-release-assay. Symbols represent mean ± SEM. (D-G) Splenocytes were stimulated for 5 hours with GP33 peptide. Contour plots are gated on CD3^+^ CD8^+^ T cells and show expression of indicated cytokines after intracellular staining. Gates were set according to unstimulated cells. (D) Contour plots show expression of IFN-γ and TNF. (E) Bar diagrams show percentage of IFN-γ^+^ cells (left), MFI of IFN-γ of IFN-γ^+^ cells (in the middle) and expression of IFN-γ and TNF double positive cells (right). (F) Contour plots show expression of IL-2 and IL-17A. (G) Bar diagrams show percentage of IL-2^+^ cells (left) and percentage of IL-17A^+^ cells (right). Statistical analysis: *p < 0.05; **p < 0.01; ***p < 0.001; ns, not significant; two-tailed unpaired Student`s *t*-test (A, E, G) or Mann-Whitney-Test (B); Error bars denote mean + SEM (A, E, G) or mean ± SEM (B, C). (A-G) Data are representative of 3 independent experiments with in total 8–9 mice per group.

Recently, it has been shown, that T-bet is able to repress several receptors associated with T cell exhaustion [35], whereas Eomes is inversely expressed in such cells. As a result, retroviral overexpression of T-bet in CD8 T cells results in the downregulation of PD-1, LAG-3 and TIM3. However, it is important to note that these markers are not only associated with exhaustion as they can be expressed transiently during an acute infection [36, 37]. Yet, haploinsufficiency for T-bet during chronic infection leads to elevated expression of these markers [35]. In line with that, GP_33_-tet^+^ CD8 T cells from *Tbx21*^*-/-*^ mice had high expression of CD39, PD-1, and LAG-3, but not TIM3 at day 8 after acute infection with LCMV WE (Fig 3A). *Tbx21*^*E/E*^ CTLs also showed high expression of CD39, PD-1, LAG-3 and, in addition, a nearly 4-fold increase in TIM3 expression (Fig 3A), which was specific for *Tbx21*^*E/E*^ mice. Functionally, this phenotype in *Tbx21*^*-/-*^ and *Tbx21*^*E/E*^ mice corresponded well with a failure of viral clearance in spleen, liver, kidney and lungs as well as significantly reduced cytotoxicity (Fig 3B and 3C). Although there was a stepwise increase in the percentage of IFN-γ producers from *Tbx21*^*+/+*^ over *Tbx21*^*-/-*^ to *Tbx21*^*E/E*^ mice (Fig 3D and 3E), CTLs from T-bet deficient genotypes produced less IFN-γ on a per cell basis (Fig 3E, middle bar diagram). Finally, loss of T-bet resulted in an increase of IL-2 production and a de-repression of IL-17 (Fig 3F and 3G). Even *Tbx21*^*E/E*^ mice showed a significant induction of GP_33_-specific IL-17, implicating that Eomes is unable to repress RORγt induction in CTLs in the absence of T-bet.

We also analyzed CD8 T cell responses against other viral epitopes (NP396 (S3A Fig) and GP276 (S3B Fig), which showed a similar pattern for cytotoxicity and cytokine production. The effects of the *Tbx21*^*E*^ allele were different in LCMV-specific CD4 T cells, which respond to the GP61 epitope. Whereas *Tbx21*^*-/-*^ mice showed a dramatic loss of IFN-γ and TNF production, this was partly rescued in *Tbx21*^*E/E*^ mice (S3C Fig). This contrast to CD8 T cells might be best explained by the lower endogenous expression of Eomes from the *bona fide Eomes* allele in CD4 T cells (Fig 1) as compared to CD8 T cells, which would evidently augment the effects of the *Tbx21*^*E*^ allele in CD4 T cells. Together, the presented data are consistent with a model, in which early induction of T-bet, and not Eomes, during acute infection with LCMV WE governs clonal expansion and functional differentiation of CTLs that leads to viral clearance.

### T-bet is intrinsically required for early expansion and differentiation of CTLs after acute LCMV WE infection

**Fig 4:**
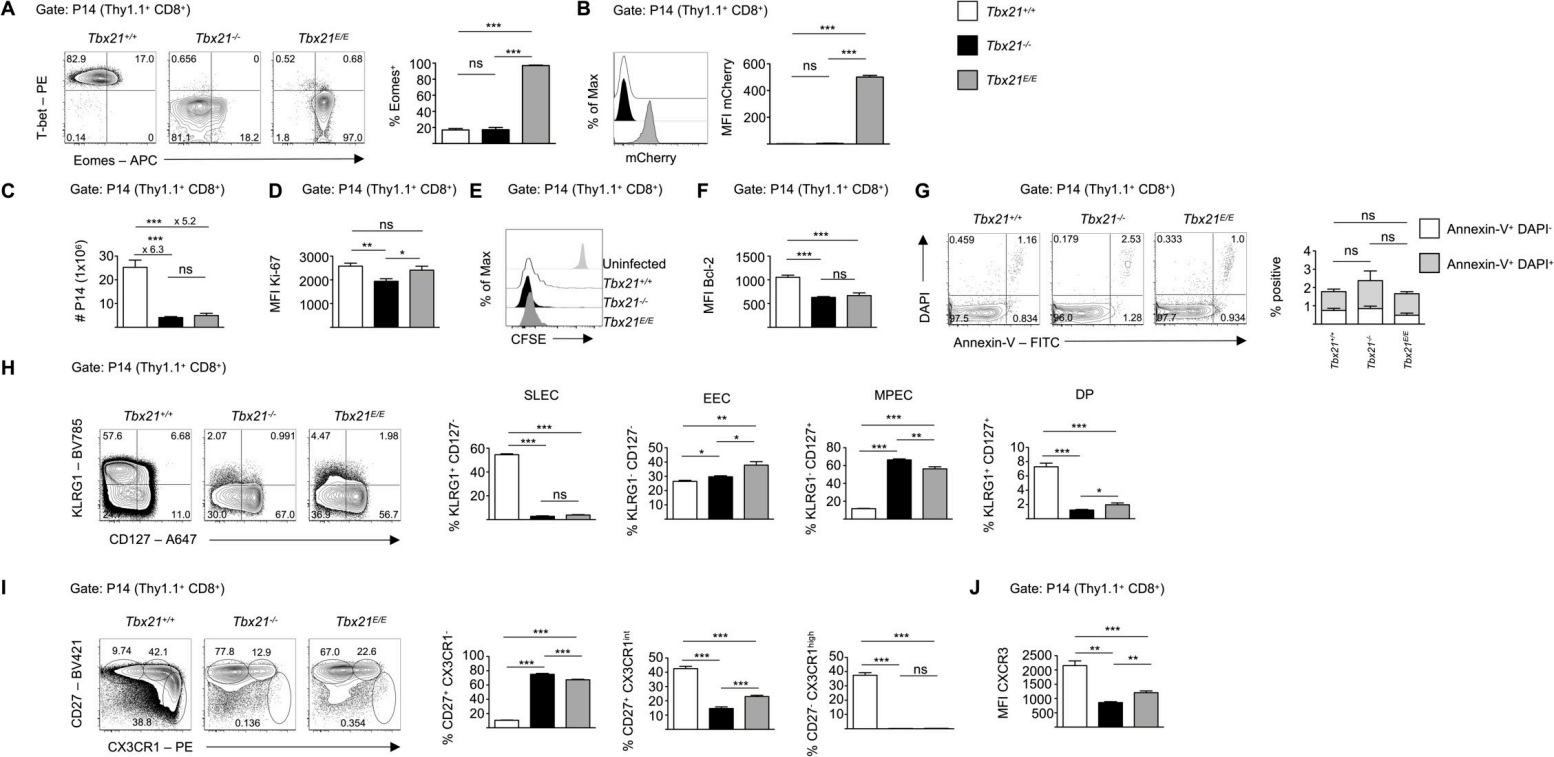
Cell-intrinsic non-redundancy of T-bet upon acute LCMV infection. 3x10^3^ CD90.1^+^ P14 CD8 T cells from *Tbx21*^*+/+*^, *Tbx21*^*-/-*^ or *Tbx21*^*E/E*^ mice were adoptively transferred into naive CD90.2^+^ C57BL/6 recipient mice. One day later, mice were infected with 200 pfu LCMV WE. If not otherwise indicated, splenocytes were analyzed by flow cytometry at day 8 post infection. (A) Contour plots show expression of T-bet and Eomes of P14 CD8 T cells as determined by flow cytometry after intranuclear staining. Quadrant gates were set according to CD44^-^ CD4^+^ T cells (T-bet^-^ Eomes^-^ cells). Bar diagram shows percentage of Eomes^+^ cells. (B) Histogram shows expression of mCherry of P14 CD8 T cells and quantification of MFI of mCherry via bar diagram. (C) Bar diagram shows absolute number (#) of P14 CD8 T cells. (D) Bar diagram shows MFI of Ki-67 of P14 CD8 T cells. (E) Histogram shows CFSE dilution of P14 CD8 T cells 96 hours post infection. Top row displays CFSE dilution of transferred P14 CD8 T cells of uninfected control group. (F) Bar diagram shows MFI of Bcl-2 of P14 CD8 T cells. (G) Contour plots show expression of DAPI and Annexin V of P14 CD8 T cells. Bar diagrams show percentage of positive cells of indicated subset. (H) Contour plots show expression of KLRG1 and CD127 of P14 CD8 T cells. Bar diagrams show percentage of SLEC (KLRG1^+^ CD127^-^), EEC (KLRG1^-^ CD127^-^), MPEC (KLRG1^-^ CD127^+^) and DP (KLRG^+^ CD127^+^) subsets. (I) Contour plots show expression of CD27 and CX3CR1of P14 CD8 T cells. Bar diagrams show frequencies of indicated subsets. (J) Bar diagram shows MFI of CXCR3 of P14 CD8 T cells. Statistical analysis: *p < 0.05; **p<0.01; ***p<0.001; ns, not significant; two-tailed unpaired Student`s *t*-test; Error bars denote mean + SEM. (A-J) Data are representative of 2 independent experiments with in total 10 mice per group.

Although the analysis of the constitutional knock-out (*Tbx21*^*-/-*^) and knock-in system (*Tbx21*^*E/E*^) had given us valuable insights on an organism-wide level, two caveats remained. First, it remained unclear whether the observed effects were CD8 T cell-intrinsic, since, beside activated CD8 T cells, various other hematopoietic cells express T-bet, which might affect CTL differentiation. Second, it is known from chronic infection models, that viral persistence has profound effects on CD8 T cell differentiation. This was important, as neither *Tbx21*^*-/-*^ nor *Tbx21*^*E/E*^ mice were free of virus on day 8 after infection. Hence, to further elucidate the CD8 T cell-intrinsic redundancy of T-bet we generated three lines of congenically marked (Thy1.1^+^) TCR-transgenic mice: P14 *Tbx21*^*+/+*^ [38], P14 *Tbx21*^*-/-*^, and P14 *Tbx21*^*E/E*^, whose CD8 T cells recognize the immuno-dominant epitope GP33 of LCMV. Next, we adoptively transferred small numbers of P14 CD8 T cells from these mice into Thy1.2^+^ wild type mice and infected them with LCMV WE (S4A Fig). Importantly, on day 8 all three groups of mice were free of virus (S4B Fig) and endogenous CTLs (CD8^+^ Thy1.1^-^) showed similar SLEC and MPEC differentiation (S4C Fig), as well as CD25 expression (S4D Fig). Hence, persistent viral infection can be excluded as a cause for functional and phenotypical differences between CTLs from P14 *Tbx21*^*+/+*^, P14 *Tbx21*^*-/-*^ and P14 *Tbx21*^*E/E*^ mice. On day 8 after infection less than 20% of the transferred CTLs from P14 *Tbx21*^*+/+*^ and *Tbx21*^*-/-*^ expressed Eomes, whereas CTLs from P14 *Tbx21*^*E/E*^ mice were uniformly Eomes^+^ (Fig 4A) and mCherry^+^ (Fig 4B). Comparable to non-TCR-transgenic CTLs, P14 *Tbx21*^*-/-*^ and P14 *Tbx21*^*E/E*^ CD8 T cells had a profound defect in clonal expansion and we therefore recovered, on average, 6- and 5-times less cells as compared to P14 *Tbx21*^*+/+*^ transferred mice, respectively (Fig 4C). Ki-67 expression, a marker for proliferating cells, showed a slightly lower, yet significant reduction in P14 *Tbx21*^*-/-*^ CTLs, whereas its expression was similar in P14 *Tbx21*^*+/+*^ and *Tbx21*^*E/E*^ CTLs (Fig 4D). However, these differences were apparently not relevant as CFSE labeled P14 CTLs from the different genotypes showed a comparable division pattern on day 4 after infection (Fig 4E). Hence, we hypothesized that the reduced accrual of T-bet deficient P14 CTLs on day 8 might be due to accelerated apoptosis. Accordingly, the anti-apoptotic molecule Bcl-2 was strongly upregulated in P14 *Tbx21*^*+/+*^ CTLs, which presumably contributed to the observed difference in recovered P14 CTLs on day 8 (Fig 4F). We could not detect an increase in apoptotic CTLs in T-bet deficient CTLs by using Annexin-V/ DAPI staining (Fig 4G), which might be due to their rapid engulfment by macrophages [39]. Collectively, the presented data suggest that Eomes cannot compensate for a loss of T-bet in rapidly dividing CTLs.

Comparable to non-TCR-transgenic T cells, SLEC differentiation was not rescued by the *Tbx21*^*E*^ allele (Fig 4H). Similar to our previous observations, T-bet deficient CTLs showed a bias towards MPECs, whereas P14 *Tbx21*^*E/E*^ CTLs had significantly less MPECs than P14 *Tbx21*^*-/-*^ CTLs (Fig 4H). Subset-specific analysis of the cytokine receptor chain CD122 revealed that in line with our previous results (S2D Fig) absence of T-bet resulted in reduced levels of CD122 in MPECs, which could not be rescued by Eomes (S4E Fig, upper row). Moreover, T-bet was essential for repression of CD25 in all subsets, a function which Eomes could partly compensate (S4E Fig, lower row). Recently, an additional approach [40] divided CD8 T cells during acute infections into three sub-populations based on the expression levels of the chemokine receptor CX3CR1 and CD27. These two markers combined generated a waterfall-like profile with CX3CR1^-^ and CX3CR1^intermediate (int)^ cells being CD27^+^, and CX3CR1^high^ cells progressively loosing CD27. This distinction was important as CX3CR1 levels correlated with homing and effector properties. Intriguingly, these authors also showed that CX3CR1^int^ were partially dependent on T-bet for their development, whereas CX3CR1^high^ cells were absolutely dependent on T-bet, suggesting further heterogeneity of CD27^+^CX3CR1^int^ CTLs. We also detected an absence of CX3CR1^high^ P14 T cells and significant reduction of CX3CR1^int^ P14 T cells after transfer of P14 *Tbx21*^*-/-*^ T cells in the spleen (Fig 4I). Transferred P14 *Tbx21*^*E/E*^ cells showed a partial rescue of CX3CR1^int^ cells, however, they also failed to differentiate into CX3CR1^high^ cells (Fig 4I). Likewise, transferred P14 *Tbx21*^*E/E*^ cells showed significantly higher expression of CXCR3 as P14 *Tbx21*^*-/-*^ cells, but never reached the high levels of P14 *Tbx21*^*+/+*^ cells (Fig 4J). This partial rescue of CX3CR1^int^ cells and CXCR3 in P14 *Tbx21*^*E/E*^ CTLs indicates a discrete functional overlap between T-bet and Eomes. Overall, these defects in clonal expansion and differentiation correlated well with immunofluorescence analysis of spleen and lymph nodes that showed reduced infiltration of adoptively transferred CD8 T cells from T-bet deficient backgrounds (S5A Fig).

### High-dimensional cytometry data analysis of CTLs reveals heterogeneity in T-box TF dependent clusters

**Fig 5:**
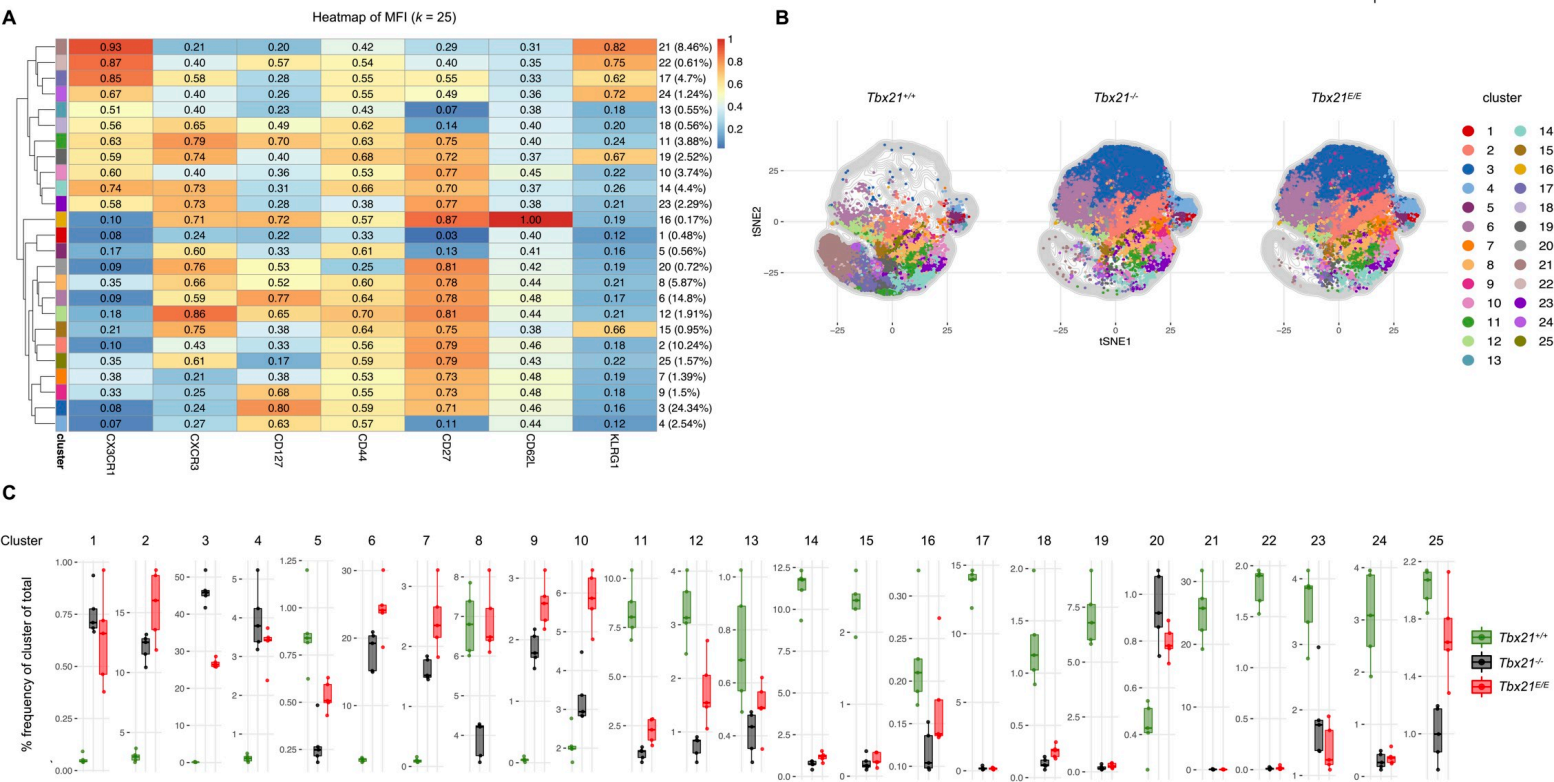
T-box TF jointly drive expansion of heterogeneous clusters of CTLs. (A) Heatmap of MFI of indicated surface markers (CX3CR1, CXCR3, CD127, CD44, CD27, CD62L, KLRG1) across the 25 cell populations obtained with FlowSOM after data was metaclustered with ConsensusClusterPlus. Dendrogram on the left represents hierarchical similarity between the 25 metaclusters. Color of heatmap represents the median of the arcsinh, 0–1 transformed marker expression calculated over cells from all samples (20,000 P14 cells per sample, n = 5 for each experimental group) with a color-coding chart on the right. Numbers denote expression values. Values in the brackets on the right indicate relative abundancy of clusters. (B) t-distributed stochastic neighbor embedding (t-SNE) map based on the arcsinh-transformed expression of surface markers (CX3CR1, CXCR3, CD127, CD44, CD27, CD62L, KLRG1) of P14 CD8 T cells. Plots represent 20,000 P14 cells, which are randomly assigned from each experimental group. Plots are stratified by indicated experimental group. Cells are color-coded according to the 25 cell populations that were obtained with FlowSOM after metaclustering the populations with ConsensusClusterPlus. (C) Frequency of indicated cluster of indicated experimental group is depicted. Box plots represent interquartile range (IQR). Horizontal line depicts median. Whiskers extend to the farthest data point within a maximum of 1.5 x IQR.

Several models of CTL differentiation based on expression of surface markers like KLRG1 and CD127 have been established. However, recent studies using single-cell RNA sequencing highlight the remarkable heterogeneity of CTLs at day 8 post infection [41, 42]. In order to obtain a higher resolution of T-bet dependent differentiation of CTLs, we adapted two recently published workflows [43, 44], which have been developed for in-depth analysis of multi-color flow cytometry data. Accordingly, we used t-distributed stochastic neighboring embedding (tSNE) to reduce the dimensionality of our 7-parameter dataset of surface markers from the experiment shown in Fig 4, which all have been implicated in the differentiation of CTLs (S5B Fig). Next, we performed hierarchical clustering on normalized median marker expression of our transferred P14 CTLs using FlowSOM [45] and ConsensusClusterPlus [46] (Fig 5A). To visualize our cluster analysis in a dimensionality-reduced fashion, obtained cluster analysis were depicted as tSNE plots (Fig 5B). At first sight, clusters were very similar distributed between T-bet deficient P14 CTLs but showed a dramatic difference in comparison to wild type P14 CTLs. As revealed by conventional gating in Fig 4H and 4I, SLECs—identified by high expression of KLRG1 and CX3CR1 and the absence of CD127 and CD27 respectively—did not develop in T-bet deficient P14 CTLs (Fig 4H and 4I). Consistently, clusters containing cells with high expression of KLRG1 and CX3CR1 as cluster 17, 19, 21, 22 and 24 were virtually absent from T-bet deficient P14 CTLs, thus validating our approach (Fig 5C).

Intriguingly, whereas conventional gating showed that the frequency of CX3CR1^int^ CD27^+^ P14 CTLs could only in part be rescued by the *Tbx21*^*E*^ allele (Fig 4I), in depth cluster analysis revealed that frequencies of clusters containing CX3CR1^int^ CD27^+^ P14 CTLs showed considerable heterogeneity. For instance, cluster 10 was around 3-times as frequent in P14 CTLs from *Tbx21*^*E/E*^ mice compared to P14 CTLs from wild type mice. In contrast, cluster 23 was hardly detectable in T-bet deficient mice. Interestingly, cluster 8, which mainly contained cells with high expression of CD27 and CXCR3—a phenotype associated with efficient re-expansion of memory CD8 T cells upon secondary challenge [47, 48]—was reduced in P14 CTLs from *Tbx21*^*-/-*^ mice, yet, in terms of frequency there was no difference between P14 CTLs from *Tbx21*^*+/+*^ and *Tbx21*^*E/E*^ mice. However, all of the more or similar abundant clusters in P14 CTLs from *Tbx21*^*E/E*^ mice were less than 6-times as frequent as in P14 wild type CTLs, suggesting a co-dependence on T-bet and Eomes for these clusters to develop or expand. Thus, timely expression of Eomes cannot replace T-bet during clonal expansion of CTLs, but Eomes and T-bet jointly drive the expansion of various CD27^+^ CTL subsets.

### T-bet intrinsically coordinates regulated effector functions in CTLs

**Fig 6:**
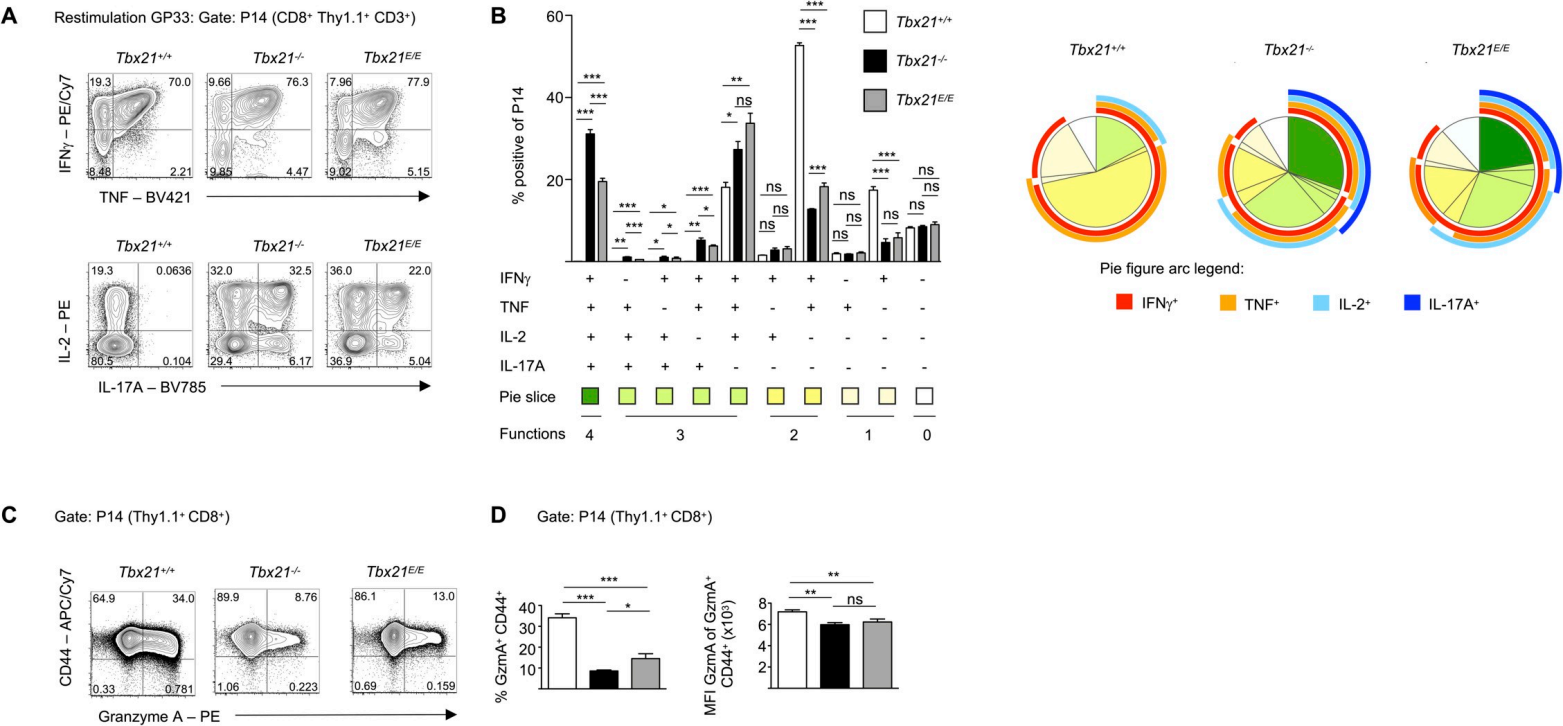
T-bet and Eomes differently regulate expression of cytokines and granzymes. (A) P14 CD8 T cells were stimulated for 5 hours with GP33 peptide. Contour plots are gated on P14 CD8^+^ CD3^+^ T cells and show expression of indicated cytokines after intracellular staining. Gates were set according to unstimulated cells. (B) Bar diagram depicts frequencies of indicated subsets determined by Boolean gating. Subsets were only considered for further analysis if the relative abundance was >0.5% in at least one of the experimental groups. Pie chart figures depict SPICE analysis of cytokine subsets of indicated groups. (C) P14 CD8 T cells were *ex vivo* isolated and expression of Granzyme A (GzmA) and CD44 was determined after intracellular staining and depicted as contour plots. (D) Bar diagram displays frequency of CD44^+^ Granzyme A^+^ P14 CD8 T cells. Histogram shows MFI of GzmA of GzmA^+^ CD44^+^ P14 CD8 T cells. Statistical analysis: *p < 0.05; **p < 0.01; ***p < 0.001; ns, not significant; two-tailed unpaired Student`s *t*-test; Error bars denote mean + SEM. (A-D) Data are representative of 2 independent experiments with in total 10 mice per group.

Next, we explored cytokine production and cytotoxic effector molecules in the different groups. Contrary to what we observed in the constitutive T-bet deficient strains, CD8 T cells from P14 *Tbx21*^*-/-*^ and P14 *Tbx21*^*E/E*^ mice showed no reduction in the cytokine level of IFN-γ^+^TNF^+^ double producers (Fig 6A). Hence, this was presumably due to extrinsic effects, e.g. insufficient CD4 T cell help, which also requires T-bet for Th1 differentiation. In addition, we discovered three times more cells that produced IL-2 in T-bet deficient P14 CD8 T cells. As expected P14 *Tbx21*^*-/-*^ CD8 T cells showed strong *de novo* production of IL-17A in around 40% of the cells, which was significantly, albeit slightly, reduced in P14 *Tbx21*^*E/E*^ CD8 T cells (Fig 6A). It also became apparent that T-bet deficient strains had a highly deregulated cytokine profile with more than half of the cells producing more than 3 cytokines and up to 30% of cells that simultaneously produced IFN-γ, TNF, IL-2 and IL-17A (Fig 6B). This wide-spread deregulation is well in line with the initial observation that T-bet is a repressor of IL-2 [49] and the later finding that it represses the development of IL-17 producing Th17 T cells [50]. Hence, these repressive actions cannot be executed by Eomes.

Activated CD8 T cells lyse infected target cells through the cooperative actions of the pore-forming protein perforin and a family of proteases termed granzymes. T-bet and Eomes together with Runx3 regulate expression of these proteins [51]. Expression of Granzyme A (GzmA) was strongly dependent on T-bet. P14 *Tbx21*^*-/-*^ CD8 T cells showed the lowest percentage of GzmA expressing cells and those cells that produced GzmA did so at a lower level per cell (Fig 6C and 6D). Similar observations were made in P14 *Tbx21*^*E/E*^ CD8 T cells (Fig 6C and 6D). In summary, this set of data illustrates that timely expression of T-bet after an acute infection governs important aspects of functional CTL differentiation.

### Early expression of Eomes *in lieu* of T-bet promotes features of exhaustion in CTLs

Besides their roles during acute infection T-bet and Eomes are also important during chronic viral infection. Functionally exhausted Eomes^high^ CD8 T cells become the dominant population as inflammation and viral replication persist during chronic infection [7]. However, it is still a matter of debate, whether high Eomes expression, ongoing viral replication or a combination of both control this dysfunctional state. To discriminate between these options, we took advantage of the fact that in our acute infection model with transferred P14 CD8 T cells mice were virus-free on day 8 but showed high expression of Eomes in P14 *Tbx21*^*E/E*^ CD8 T cells in contrast to P14 *Tbx21*^*+/+*^ CD8 T cells, which, reciprocally, showed high expression of T-bet. Hence, we further analyzed the phenotype of our transferred P14 CD8 T cells, focusing on inhibitory receptors and transcription factors, which are associated with exhausted CD8 T cells.

**Fig 7:**
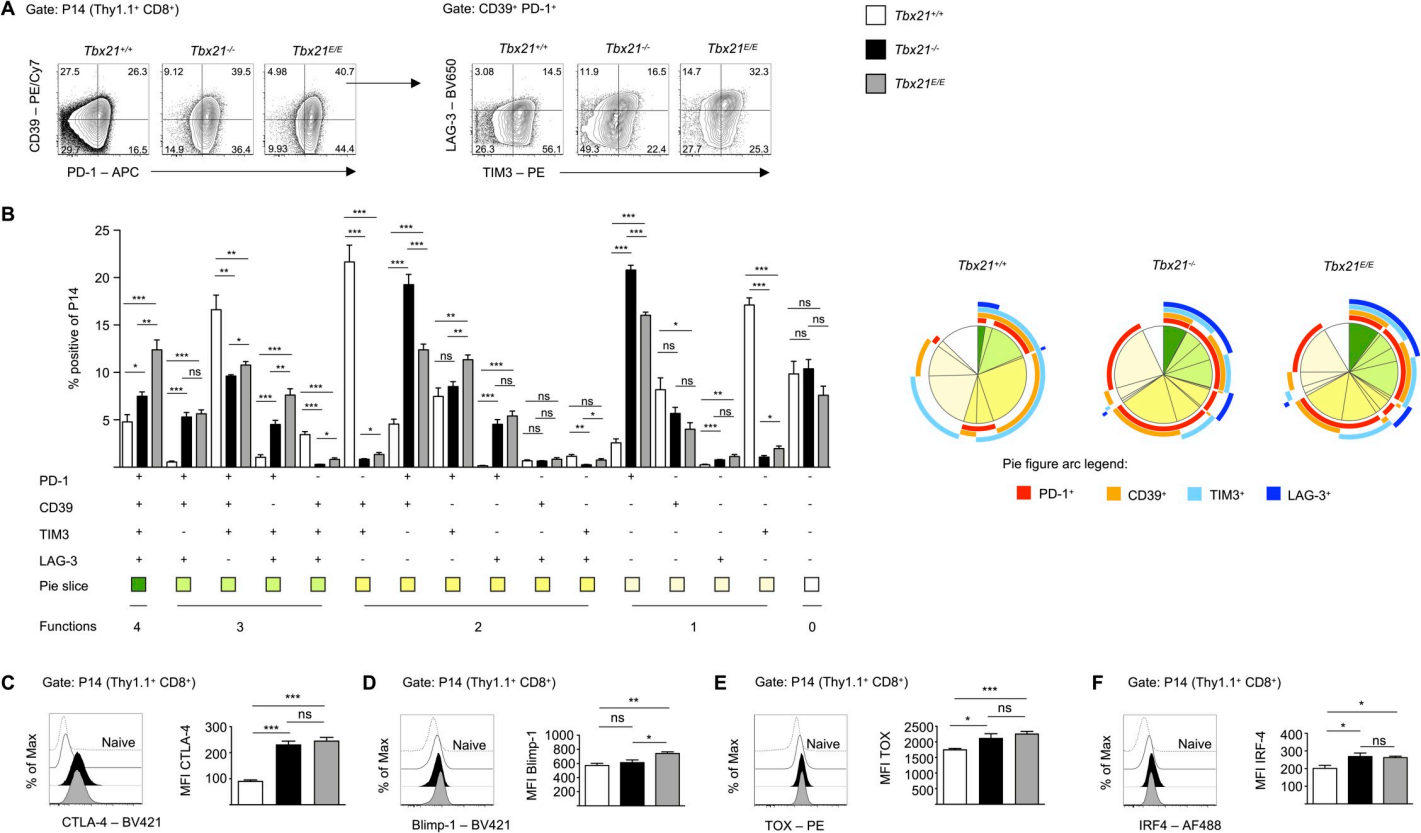
High expression of Eomes promotes features of exhaustion upon acute LCMV infection. (A) Contour plots (left side) show expression of CD39 and PD-1 of P14 CD8 T cells. Contour plots on the right show expression of LAG-3 and TIM3 of CD39^+^ PD-1^+^ P14 CD8 T cells. Gates were set according to CD44^-^ CD8 T cells. (B) Bar diagram shows frequencies of indicated subsets determined by Boolean gating. Subsets were only considered for further analysis if the relative abundance was >0.5% in at least one of the experimental groups. Pie chart figures depict SPICE analysis of inhibitory receptors of indicated groups. (C-F) Dashed lines serve as control and refer to naïve (CD44^-^) CD8 T cells. (C) Histogram shows expression of CTLA-4. Bar diagram shows MFI of CTLA-4 of P14 CD8 T cells. (D) Histogram shows expression of Blimp-1 of P14 CD8 T cells. Bar diagram shows MFI of Blimp-1 of P14 CD8 T cells. (E) Histogram shows expression of TOX of P14 CD8 T cells. Bar diagram shows MFI of TOX of P14 CD8 T cells. (F) Histogram shows expression of IRF-4 of P14 CD8 T cells. Bar diagram shows MFI of IRF-4 of P14 CD8 T cells. Statistical analysis: *p < 0.05; **p<0.01; ***p<0.001; ns, not significant; two-tailed unpaired Student`s *t*-test; Error bars denote mean + SEM. (A-F) Data are representative of 2 independent experiments with in total 10 mice per group.

As outlined above, CD39, PD-1, LAG-3, TIM3 and CTLA-4 are inhibitory receptors, that can be transiently expressed during acute infections, but whose prolonged and simultaneous expression on virus-specific CD8 T cells during chronic infection identifies cells that are functionally exhausted [23, 36, 52]. Intriguingly, more than half of P14 CTLs from *Tbx21*^*+/+*^ mice expressed either CD39 or TIM3 or both and this expression pattern was hardly detectable in T-bet deficient P14 CTLs (Fig 7A and 7B). In contrast, we saw a stepwise increase in the percentage of P14 CTLs that were simultaneously positive for PD-1, CD39, TIM3 and LAG-3 from *Tbx21*^*+/+*^ over *Tbx21*^*-/-*^ to *Tbx21*^*E/E*^ mice (Fig 7A and 7B). Moreover, the immune checkpoint CTLA-4, that inhibits co-stimulation via competition with CD28, was strongly induced in P14 CTLs from T-bet deficient mice (Fig 7C). The fact, that *Tbx21*^*E/E*^ P14 CTLs had a higher percentage of PD-1^+^CD39^+^TIM3^+^LAG-3^+^ cells than P14 *Tbx21*^*-/-*^ CTLs, clearly indicates that this phenotype cannot solely be attributed to the absence of T-bet, but rather by the additional direct or indirect actions of the *Tbx21*^*E*^ allele. Thus, we gauged the effects of our *Tbx21*^*E*^ allele on other TFs that contribute to the phenotype of exhausted T cells. Previously, it has been shown that Blimp-1 regulates expression of inhibitory receptors [53, 56]. In line with our results, we observed a significant increase of Blimp-1 expression [53] in P14 *Tbx21*^*E/E*^ CTLs (Fig 7D). Moreover, we detected similarly elevated levels of TOX [54] (Fig 7E) and IRF4 [55] (Fig 7F), two recently identified regulators of exhausted T cells, in P14 *Tbx21*^*-/-*^ and P14 *Tbx21*^*E/E*^ CTLs as compared to P14 *Tbx21*^*+/+*^ CTLs. This would argue that TOX and IRF4 are primarily induced upon loss of T-bet and not via expression of Eomes. Collectively, over-expression of Eomes at an early stage of acute viral infection promotes some features of an exhausted phenotype that is independent of viral persistence.

## Discussion

Here we have tested whether the synchronized induction of Eomes in T-bet deficient mice can compensate for the loss of T-bet during an acute viral infection. Combining two different model systems, we show that T-bet is intrinsically required for canonical effector cell differentiation and clonal expansion of CTLs during acute infection with LCMV WE. Moreover, we demonstrate that T-bet non-redundantly limits IL-2 production and inhibits aberrant expression of IL-17A in LCMV WE-specific CTLs. In summary, we can conclude that T-bet, has unique and important functions during acute viral infection, which cannot be executed by its paralog Eomes.

The biological complexity of multicellular organism has been facilitated by genome duplication. This allowed for a process of diversification and selection of new genes and functions that provided the organism with many advantages under evolutionary pressure [57]. The T-box TF family is a prime example of such processes. Hence, it is not surprising that the two paralogs T-bet and Eomes, despite their high degree of homology, have only very limited functional overlap *in vivo*, as we have shown here. Nevertheless, it remains striking that T-bet and Eomes share the highest degree of homology within their DNA-binding sequence; i.e. the T-box (ca. 74%). Here, it is important to point out, that T-box TFs utilize a variety of mechanisms to establish a gene expression program. Among these are context-dependent dimerization, binding of co-factors and sequestration of alternative transcription factors as well as epigenetic modification through recruitment of histone modifying enzymes [58]. Moreover, T-box TFs can compete for promoter binding and, once bound, can exhibit either activation or repression activities [59]. Thus, it is difficult to predict the actions of T-bet and Eomes solely based on their respective consensus DNA-binding motif, which is conspicuously similar. Relating to this, it is of note that T-bet and Eomes show only 29% similarity in their N- and C-terminus [3]. These regions will most likely contribute to the observed functional differences of T-bet and Eomes and studies are under way to test this prediction. In line with this, a recent publication that compared CHIPseq data from T-bet and Eomes in CD8 CTLs demonstrated that Eomes bound only 1292 out of 7534 (17.15%) T-bet target genes [34]. This implicates that more than 80% of the transcriptional signature of T-bet is unique.

The population size of T cells during infection is regulated by a dynamic balance between proliferation and survival, which sequentially results in massive expansion, contraction and memory maintenance of small numbers of antigen-specific T cells [60]. Here, we have extended previous reports by showing that T-bet was essential for early expansion of CTLs and that this defect in *Tbx21*^*-/-*^ mice could not be overcome by the timely expression of Eomes in *Tbx21*^*E/E*^ mice. We noted a significant reduction in the expression of the anti-apoptotic molecule Bcl-2 in T-bet deficient strains, which is therefore most likely regulated, either directly or indirectly, by T-bet. In contrast, we detected only mild differences in expression of the proliferation marker Ki-67, which is further supported by similar CFSE division profiles in the three genotypes. Hence, it is most likely that T-bet is important for survival of proliferating CTLs during acute infection. This is further corroborated by data from Prier et al. [61], which showed that *Tbx21*^*-/-*^ CD8 T cells have no early expansion defect following Influenza A virus infection and, in line with our data, this study also did not detect a compensatory increase of Eomes in *Tbx21*^*-/-*^ CD8 T cells after adoptive transfer. Actions of T-bet on survival could be regulated via cytokine responsiveness as T-bet deficient strains showed reduced expression of CD122 in both models. CD122 represents the IL-2Rβ chain, which is important for IL-2 and IL-15 signaling, which in turn can regulate pro- and anti-apoptotic molecules. Although previous experiments have indicated that both, Eomes and T-bet can transactivate CD122 [16] when genetically introduced into T cells, CTLs from P14 *Tbx21*^*E/E*^ mice did not rescue the reduced expression of CD122 in CTLs from P14 *Tbx21*^*-/-*^ mice. This difference can be explained by the fact that in contrast to our precisely controlled knock-in model earlier studies used retroviral overexpression systems [16], which often result in supraphysiological doses of TFs. Hence, maximum expression of CD122 during acute infection requires the cooperative actions of T-bet and Eomes. Intriguingly, T-bet deficient strains showed clear evidence of heightened IL-2 signaling. First, CTLs from T-bet deficient mice expressed significantly more CD25, which represent the high-affinity IL-2Rα chain [62]. Second, CD25 can be up-regulated by IL-2 and correspondingly we detected abundant IL-2 production in T-bet deficient CTLs. Prolonged IL-2 signaling, especially in the context of inflammation, drives terminal effector differentiation, whereas curtailed IL-2 signaling during infection favors CD8 memory formation [63]. However, this heightened IL-2 signaling in T-bet deficient strains was not sufficient to rescue SLEC formation in CTLs from P14 *Tbx21*^*E/E*^ mice arguing that IL-2 must act jointly with T-bet to drive SLEC formation. IL-2, in part, acts via the transcriptional regulators Id2 [64] and Blimp-1 [63, 65]. Consistent with a previous study, Blimp-1 expression levels did not differ between WT and *Tbx21*^*-/-*^ mice [65], whereas *Tbx21*^*E/E*^ mice did upregulate Blimp-1 significantly. This was, however, not sufficient to compensate for the loss of T-bet. In addition to its role during differentiation, early studies have also shown that activation-induced cell death is strongly promoted by IL-2 [66]. Hence, it will be of further interest to determine, whether the heightened IL-2 signaling in T-bet deficient strains contributes to their failure to accumulate at day 8 of infection.

Another important aspect of our findings is the different phenotype of LCMV-specific CTLs in our two model systems. Whereas GP_33_-tet^+^ T cells in *Tbx21*^*-/-*^ und *Tbx21*^*E/E*^ failed to produce the high amounts of IFN-γ and TNF per cell as seen in *Tbx21*^*+/+*^ mice, this difference was not observed in the adoptive transfer system, where P14 *Tbx21*^*+/+*^, P14 *Tbx21*^*-/-*^ and P14 *Tbx21*^*E/E*^ CTLs all produced high amounts of IFN-γ and TNF. These results are most likely mitigated by extrinsic effects of T-bet deficiency on CTL differentiation in *Tbx21*^*-/-*^ und *Tbx21*^*E/E*^ mice. In fact, T-bet is not only expressed by CD8 T cells but also by other lymphocytes and even myeloid cells, that all shape the immune response against LCMV. T-bet was initially discovered as the lineage-defining TF of CD4 Th1 cells [2], as it was able to instruct abundant production of IFN-γ. Although CD4 T cell help is dispensable for short-term acute LCMV infection, LCMV-specific CD4 T cells will get activated during acute LCMV WE infection and provide T cell help. Interestingly, timely expression of Eomes in LCMV-specific CD4 T cells from *Tbx21*^*E/E*^ mice rescued the frequency of non-TCR-transgenic IFN-γ producers as compared to *Tbx21*^*-/-*^ mice (S3C Fig), however they produced less IFN-γ per cell as LCMV-specific CD4 T cells from *Tbx21*^*+/+*^ mice. It is therefore unlikely, that these small differences in the CD4 compartment can explain the differences in CTL responses in our two models. Hence, it will be of future interest to investigate and compare cell types, that usually express T-bet (e.g. B cells, NKT, group 1 innate lymphoid cells, dendritic cells) in *Tbx21*^*E/E*^ mice to understand their contribution to the significant defect in IFN-γ production in CTLs from T-bet deficient strains. Another interesting feature of the adoptive transfer system was the revelation that certain aspects of T-bet could be partially rescued by Eomes; e.g. expression of CD25 (Fig 4E), CXCR3 (Fig 4I) and frequency of CD27^+^CX3CR1^int^ (Fig 4J) in P14 *Tbx21*^*E/E*^ CTLs. However, this never reached the levels seen in P14 *Tbx21*^*+/+*^ CTLs and in general, P14 *Tbx21*^*E/E*^ CTLs were more akin to P14 *Tbx21*^*-/-*^ CTLs. We would therefore be very cautious to attribute a biological significance to the partial overlap P14 *Tbx21*^*E/E*^ CTLs share with P14 *Tbx21*^*+/+*^ CTLs.

Finally, we used our novel model to uncouple the effects of elevated Eomes expression and ongoing viral replication on the phenotype of activated CD8 T cells. Previous reports have shown that Eomes and T-bet are differentially linked to the exhausted phenotype of CD8 T cells in chronic viral infections [7] or tumors [34]. In such disease states T-bet and Eomes are reciprocally expressed and Eomes expression increases with time and ongoing viral replication or tumor burden, respectively. Moreover, Eomes expression correlates with higher expression of inhibitory receptors, e.g. PD-1, LAG-3, CD160, 2B4, TIM3, TIGIT, CTLA-4, and impaired cytokine production, which in concert define the state of exhaustion [24]. Additionally, the HMG-box transcription factor TOX was recently identified as an important regulator of exhausted T cells [54, 67], and its conditional deletion resulted in reduced expression of inhibitory receptors and Eomes as well as improved effector functions. We extend this set of data by showing that, even in the absence of viral replication, uniform expression of Eomes in P14 CTLs from *Tbx21*^*E/E*^ mice fosters a partially exhaustion-like phenotype, which was characterized by the highest frequency of CD39^+^PD-1^+^LAG-3^+^TIM3^+^ P14 CTLs. Moreover, T-bet also represses the inhibitory T cell checkpoint CTLA-4 [68], which is highly expressed in T-bet deficient P14 CTLs (Fig 7C), irrespective of Eomes levels. Of particular interest was the expression pattern of PD-1 and LAG-3, which were simultaneously expressed in a significant proportion of T-bet deficient CTLs in both models (Figs 3A, 7A and 7B). The dual blockade of LAG-3 and PD-1 shows synergistic effects in models of chronic LCMV infection [36] and tumors [69] and has resulted in several ongoing clinical trials [70]. Data from the transfer system in Fig 7A and 7B clearly illustrates that T-bet represses PD-1 but that Eomes can induce LAG-3 as P14 *Tbx21*^*E/E*^ CTLs show the highest expression levels for LAG-3. Similar results were also observed in our non-TCR-transgenic system (Fig 3A). In summary, we conclude that T-bet and Eomes presumably regulate different aspects of exhaustion in CTLs, which would be in line with recent findings [23, 71]. It is important to note that Eomes is not only associated with exhaustion but also an important transcription factor in memory T cells. Eomes expression gradually increases after an acute LCMV infection and as a result, Eomes deficient CTLs have a competitive disadvantage and do not contribute sufficiently to the memory pool. In the future, it will therefore be of interest to determine, whether the enforced expression of Eomes in *Tbx21*^*E/E*^ CTLs accelerates the establishment of a memory program at the cost of an acute effector program. Hence, it is conceivable that *Tbx21*^*E/E*^ CD8 T cells harbor an increased memory population (S1C Fig) after an acute LCMV infection, which potentially still fails to expand upon re-challenge due to the absence of T-bet.

Despite its effect on inhibitory receptors and proliferation T-bet deficiency did not lead to the full spectrum of exhaustion, as transferred P14 *Tbx21*^*-/-*^ and *Tbx21*^*E/E*^ T cells showed no impairment in IFN-γ or TNF production. Most importantly, IL-2 production, which is lost early during exhaustion, was much higher in T-bet deficient CTLs, which is well in line with T-bet’s ability to repress IL-2. T-bet was also absolutely required to repress IL-17 production in CTLs, which was strikingly deregulated in T-bet deficient strains. Hence, heightened expression of Eomes might explain some features of exhaustion but must act in concert with other factors during chronic infections.

In conclusion, the presented findings underline the non-redundancy of T-bet in acute viral infections and emphasize the necessity for further research to mechanistically dissect the transcriptional activities of T-bet and Eomes. This will help to identify TF-specific targets that could be harnessed therapeutically in the fight against chronic infections and cancer as well as amelioration of immune-mediated diseases.

## Material and methods

### Ethics statement

All animal experiments were approved and are in accordance with the local animal care committees of the University of Freiburg and the Regierungspräsidium Freiburg (approval number G-19/64).

### Mouse strains

*Tbx21*^*-/-*^, *Eomes*^*Gfp/+*^, *T-cre Eomes*^*flox/flox*^ (*Cre* transgene is driven by the T (*Brachyury*) promotor that induces a pan-mesodermal loss of Eomes, including all hematopoietic cells) and P14 TCR tg mice on a C57BL/6 background were bred locally. C57BL/6 mice were purchased from Janvier Laboratories (LeGenest St-Isle, France). Mice of both sex between 8 to 12 weeks of age were used.

### Generation of the *Tbx21*^*Eomes-P2A-mCherry/+*^ (*Tbx21*^*E/+*^) mouse

Targeting of the *Tbx21* locus for the generation of the *Tbx21*^*Eomes-P2A-mCherry/+*^ allele was performed by TALEN-enhanced homologous recombination [27] in ES cells. Two TALENs were directed against sequences close to exon 1 within the first intron of the *Tbx21* gene. A targeting vector was generated that contains 500bp of 5' and 3' homology regions surrounding exon 1 of *Tbx21*, a Eomes-P2A-mCherry cassette introduced at the translational start site (thereby deleting coding sequences of exon 1), and a LoxP-flanked neomycin resistance cassette. Linearized targeting vector together with TALEN-coding vector were electroporated into CCE ES cells using Amaxa 4D Nucleofector protocol for Mouse Embryonic Stem Cells and neomycin resistant ES cell colonies were screened by genomic PCR for correct integration into the gene locus. Generation of chimeras was performed by laser-assisted injections at morula stage (Transgenic Core Facility, Max-Planck-Institute Dresden) and F1 progeny and subsequent generations were genotyped by PCR. The strain was maintained by backcrossing to C57BL/6 mice. For experiments, 8 to 12 weeks old mice, which were backcrossed 6–10 generations to C57BL/6, were used. For steady state analysis, littermates derived from mating *Tbx21*^*E/+*^ with *Tbx21*^*E/+*^ mice were used.

### *In vitro* stimulation assay of splenocytes

Splenocytes were isolated from *Tbx21*^*+/+*^, *Tbx21*^*-/-*^, *Tbx21*^*E/E*^ and *T-cre Eomes*^*flox/flox*^ mice. Next, 0.5x10^6^ splenocytes were cultured in flat-bottom 24-well plates (Sigma-Aldrich, St.Louis, Missouri, US) in 1 ml complete medium (RPMI 1640 supplemented with 10% FCS, 10 mM Hepes, 55 μM 2-Mercaptoethanol, 2 mM L-Glutamine, 100 U/ml Penicillin and 100 μg/ml Streptomycin) and stimulated with or without soluble anti-CD3 (1μg/mL, 145-2C11, BioLegend, San Diego, US) with or without IL-12 (10ng/mL, PeproTech, Hamburg, Germany) and analyzed after 48 or 96 hours.

### Flow cytometry analysis and cell sorting

Single cell suspensions from spleen, lymph nodes and thymus were obtained by meshing spleens, thymocytes and axillary and inguinal lymph nodes through 70 μm cell strainers (BD Biosciences, San Jose, CA, USA). Next, single cell suspensions were depleted of erythrocytes by incubation with red cell lysis buffer. Afterwards, cells were incubated on ice for 30 min with an anti-CD16/CD32 antibody for FC receptor blocking in PBS (Ca2^+^ and Mg2^+^-free) supplemented with 2 mM EDTA and 2% FCS. After a washing step, single cells were stained with murine fluorescence-labelled antibodies for 30 to 60 min on ice in the dark.

The following conjugated antibodies were obtained from BioLegend, Thermo Fisher Scientific (Waltham, Massachusetts, US) and BD Biosciences: CD4 (RM4-5), CD8α (53–6.7), TCRβ (Η57–597), CD90.1 (Thy1.1, OX-7), CD122 (TM-β1), CD25 (PC61), CD44 (IM7), CD62L (MEL-14), PD-1 (29.F1A12), B220 (RA3-6B2), KLRG1 (2F1/KLRG1), CD127 (A7R34), CXCR3 (CXCR3-173), CXCR4 (L276F12), CD39 (Duha59), TIM3 (B8.2C12 in PerCP/Cy5.5 and RMT3-23 in PE), LAG-3 (C9B7W), CD27 (LG.3A10), CX3CR1 (SA011F11) and DAPI.

T-bet (4B10), Eomes (Dan11mag), CTLA-4 (UC10-4B9), GranzymeA (3G8.5), TOX (TXRX10), Blimp-1 (5E7), IRF4 (IRF4.3E4), Ki-67 (B56) and Bcl-2 (100) were stained by using the FoxP3 transcription factor staining buffer set (Thermo Fisher Scientific) according to manufacturer instructions. In order to stain GFP-reporter mice in combination with transcription factors (TFs), cells were fixated with Cytofix/Perm (BD Biosciences) for 20 min after surface staining, washed twice in wash buffer (BD Biosciences) and incubated with an anti-GFP antibody (clone 3E6, Thermo Fisher Scientific) for 1 hour. After washing, cells were fixated overnight in FoxP3 transcription factor staining buffer set (Thermo Fisher Scientific), washed twice and TFs were stained in FoxP3 transcription factor Perm/Wash buffer for 2 hours. For intracellular cytokine staining, 10^6^ splenocytes were stimulated with 10^−7^ M GP33,—GP276,—NP396 or GP61 peptide for 5 hours in the presence Brefeldin A (20μg/mL) at 37 °C. After surface staining, cells were fixated using Cytofix/Perm (BD Biosciences) for 20 min. Then, cells were washed two times with wash buffer (PBS supplemented with 0.5% saponine and 5% FCS) and intracellularly stained with antibodies against CD3 (17A2), IL-2 (JES6-5H4), IFNγ (XMG1.2), TNF (MP6-XT22) and IL-17A (TC11-18H10.1) and washed two times with aforementioned wash buffer. In order to obtain virus-specific CD8^+^ T cells, lymphocytes were stained with H-2 D^b^ GP_33_-streptamer (IBA Gmbh, Göttingen, Germany) according to manufacturer instructions. For Annexin V staining, cells were stained with surface antibodies followed by an Annexin V-FITC (Biolegend) labeling in Annexin V buffer (Biolegend) for 15 min at room temperature and measured within one hour by flow cytometry. DAPI (3μM) was added immediately before acquisition. Flow cytometry was performed with a LSRFortessa (BD Bioscience) flow cytometer and analysed using FlowJo software V9 (Treestar, Ashland, Oregon, US). Pie charts were generated using SPICE software Version 6.0.

### Virus and infections

LCMV WE was grown on L929 cells. Mice were inoculated with 200 plaque forming units (pfu) via intravenous application.

### ^51^Chromium-release assay

Cytolytic activity of CD8^+^ T cells was determined using a standard ^51^Chromium release assay as previously described [72, 73]. In brief, GP33, GP276 and NP396 peptide-loaded EL-4 cells were used as target cells and incubated with different concentrations of splenocytes as effector cells for 5 hours at 37 °C. Duplicate wells were assayed for each effector-target ratio and percentages of specific lysis were calculated.

### Viral titers

Viral titers were determined using a standard focus-forming assay as previously described [72, 73].

### Adoptive transfer experiments

P14 cells expressing a transgenic (tg) TCR specific for the LCMV glycoprotein GP_33_ in the context of H2-D^b^ were isolated from spleens of P14 TCR tg *Tbx21*^*+/+*^, *Tbx21*^*-/-*^ and *Tbx21*^*E/E*^ mice on a CD90.1 (Thy1.1) background using the Dynabeads Untouched Mouse CD8 Cell Kit (Thermo Fisher Scientific) according to manufacturer instructions (Purity >90%). 3x10^3^ P14 cells (DAPI^-^ CD8^+^ TCRVα2^+^ CD90.1^+^) were adoptively transferred via intravenous injection into C57BL/6 recipient mice on a CD90.2 (Thy1.2) background and infected with 200 pfu LCMV WE one day later.

### Adoptive transfers of CFSE labeled P14 cells

After enrichment of P14 CD8 cells of the different genotypes, cells were washed with PBS and 1x10^7^ cells/ml were labeled with 5μM CFSE (Biolegend) in PBS for 10 min at 37 ^°^C. Cells were washed twice in complete RPMI medium and 1x10^6^ P14 CD8 T cells were immediately transferred into recipient wild type mice (Thy1.2^+^) one day before infection with 200 pfu LCMV WE. CFSE dilution of P14 CD8 T cells was analyzed by flow cytometry four days after infection.

### Immunofluorescence

Spleens and inguinal lymph nodes were removed and cleaned by washing in ice-cold PBS. A piece of the tissue was fixed in 4% PFA on ice for 2h. Afterwards the tissue was washed in ice-cold PBS before rehydrating the tissue in 30% (w/v) sucrose phosphate buffer overnight. Tissues samples were embedded in O.C.T. Compound (Tissue-Tek) and snap-frozen in liquid nitrogen. Cryostat sections of 6 μm thickness were collected on frosted glass slides. Slides were rehydrated in PBS and blocked with 5% BSA (Sigma-Aldrich) for 60 min at room temperature. Samples were incubated with fluorescein isothiocyanate conjugated monoclonal anti-mouse CD90.1/Thy1.1 antibody (clone OX-7, Biolegend, San Diego, CA) and allophycocyanin conjugated monoclonal anti-mouse CD45R/B220 antibody (clone RA3-6B2, Biolegend, San Diego, CA), each diluted 1:500 in 5% BSA for 60 min at room temperature. Slides were washed with PBS and mounted with mounting medium (PermaFlour, Thermo Scientific, Waltham, MA) and each section was visually inspected. Images of representative sections were captured under an Axioplan 2 fluorescence microscope at 20x magnification using an AxioCam camera and Axiovision LE software Rel. 4.9 (all Axio devices were from Zeiss, Oberkochen, Germany).

### High-dimensional flow cytometry data analysis

Analysis was adapted from two published workflows [43, 44]. In brief, to perform in-depth analysis of polychromatic flow cytometry data, flow cytometry standard (FCS) 3.0 files were imported into FlowJo Version 9. Next, P14 cells (Thy1.1^+^ CD8^+^) were downsampled to 20,000 cells per sample. After that, data was imported into Cytobank (Santa Clara, CA, US) to determine an appropriate cofactor for arcsinh-transformation of data. Next, data was loaded into R (V3.4.1) and arcsinh-transformed and further analyzed in R as outlined: Data was transformed to values from 0 to 1 and t-distributed stochastic neighbor embedding (t-SNE) was run (perplexitiy = 100, interations = 2000) on surface markers (CX3CR1, KLRG1, CXCR3, CD127, CD44, CD27, CD62L) of P14 cells (20,000 P14 cells per sample, n = 5 samples per experimental group, total of 300,000 P14 cells). Next, P14 cells were hierarchically clustered using FlowSOM [45] and ConsensusClusterPlus [46] based on the mean fluorescence expression (MFI) of aforementioned surface markers transformed to values from 0 to 1. Thus, self-organizing maps (SOM) were generated and after that SOM codes were metaclustered with the ConsensusClusterPlus package (maxK = 25) and MFI and frequency of obtained clusters were depicted as heatmap using the pheatmap package in R.

### Statistical analysis

If not otherwise indicated, p value of data sets was determined by unpaired two-tailed Student`s t-test with 95% confidence interval. If indicated, data was Welch-corrected prior unpaired two-tailed Student`s t-test. All statistical tests were performed with Graph Pad Prism V4 software (Graph Pad Software, La Jolla, CA). (*p < 0.05; **p < 0.01 and ***p < 0.001; ns, not significant).

## Supporting information

S1 Fig (A-D) Lymphocytes were isolated from the thymus, inguinal and axillary lymph nodes and spleen from naive 8–12 weeks old *Tbx21*^*+/+*^, *Tbx21*^*E/+*^ and *Tbx21*^*E/E*^ mice (littermates) and analyzed by flow cytometry. (A) Bar diagram shows absolute (#) cell number of lymphocytes isolated from indicated organs and groups. (B) Contour plots show CD4 and CD8 expression of thymic cells. Bar diagram shows frequencies of CD4^+^ CD8^-^ (CD4 SP) cells, CD4^+^ CD8^+^ (DP) cells, CD4^-^ CD8^+^ (CD8 SP) and CD4^-^ CD8^-^ (DN) cells of indicated groups. (C) Contour plots and quantification of lymphocyte subsets via bar diagrams of frequencies of B cells (B220^+^ TCRβ^-^), T cells (B220^-^ TCRβ^+^), CD4^+^ T cells (CD4^+^ TCRβ^+^), CD8^+^ T cells (CD8^+^ TCRβ^+^), T_EM_ cells (CD8^+^ CD44^+^ CD62L^-^), T_CM_ cells (CD8^+^ CD44^+^ CD62L^+^) and CD4^+^ CD25^+^ regulatory T cells (from top to bottom) isolated from the spleen of indicated groups. (D) Contour plots and quantification of lymphocyte subsets via bar diagrams of frequencies of B cells (B220^+^ TCRβ^-^), T cells (B220^-^ TCRβ^+^), CD4^+^ T cells (CD4^+^ TCRβ^+^), CD8^+^ T cells (CD8^+^ TCRβ^+^), T_EM_ cells (CD8^+^ CD44^+^ CD62L^-^), T_CM_ cells (CD8^+^ CD44^+^ CD62L^+^) and CD4^+^ CD25^+^ regulatory T cells (from top to bottom) isolated from the inguinal and axillary lymph nodes of indicated groups. (E) Histograms show representative MFIs of T-bet of indicated lymphocyte subsets isolated from splenocytes of 8–12 weeks old *Tbx21*^*+/+*^ mice. (F) Histograms show representative MFIs of mCherry of indicated lymphocyte subsets isolated from splenocytes of 8–12 weeks old *Tbx21*^*E/E*^ mice. Statistical analysis: *p < 0.05; **p < 0.01; ***p < 0.001; ns, not significant; two-tailed unpaired Student`s *t*-test; Error bars denote mean + SEM. (A-D) Data are representative (contour plots) or cumulative (bar diagrams) from n = 4–6 mice per group from 2 independent experiments with n = 2–4 mice per group and experiment.
(TIF)

S2 Fig *Tbx21*^*+/+*^, *Tbx21*^*-/-*^ and *Tbx21*^*E/E*^ or *Eomes*^*+/+*^ and *Eomes*^*Gfp/+*^ mice were infected with 200 pfu LCMV WE. At day 8 p.i. splenocytes of indicated groups were analyzed by flow cytometry. (A) Bar diagram (left) shows absolute (#) number of splenocytes. Bar diagram (middle) shows frequency of CD4^+^ T cells. Right bar diagram shows absolute (#) numbers of CD4^+^ T cells. (B) Contour plots (left) show expression of H-2D^b^ GP_33_-tetramer and GFP (Eomes). Bar diagram shows frequency of GFP^+^ (Eomes^+^) cells of GP_33_-tet^+^ cells. Contour plots (right) show expression of T-bet and Eomes after intranuclear staining of GP_33_-tet^+^ CD8^+^ cells. (C) Contour plots show expression of T-bet and Eomes of CD8^+^ CD44^+^ T cells as determined by flow cytometry after intranuclear staining. Quadrant gates were set according to CD44^-^ CD4^+^ T cells (T-bet^-^ Eomes^-^ cells). Bar diagram shows percentage of Eomes^+^ cells. (D) Contour plots show expression of CD122 and CD127 of CD4^+^ T cells (left) or of GP_33_-tet^+^ CD8^+^ cells (right). Bar diagrams show MFI of CD122 of CD127^-^ (left) or CD127^+^ (right) GP_33_-tet^+^ CD8^+^ cells. Statistical analysis: *p < 0.05; **p < 0.01; ***p < 0.001; ns, not significant; two-tailed unpaired Student`s *t*-test; Error bars denote mean + SEM. (A, C, D) Data are representative of 3 independent experiments with in total 8–9 mice per group. (B) Data are representative from 2 independent experiments with n = 3 mice per experimental group.
(TIF)

S3 Fig *Tbx21*^*+/+*^, *Tbx21*^*-/-*^ and *Tbx21*^*E/E*^ mice were infected with 200 pfu LCMV WE. At day 8 p.i. splenocytes were analyzed by flow cytometry. Next, splenocytes were stimulated for 5 hours with indicated peptide. Contour plots are gated on CD3^+^ CD8^+^ T cells (A, B) or CD3^+^ CD4^+^ T cells (C) and show expression of indicated cytokines after intracellular staining. Gates were set according to unstimulated cells. (A) Contour plots show expression of IFN-γ and TNF (left) or IL-2 and IL-17A (right) of indicated experimental groups. Bar diagrams show frequencies of indicated subsets. Cytolytic activity of CD8^+^ T cells was determined on NP396-peptide loaded EL-4 target cells in a 5-hour-^51^Cr-release-assay. Symbols represent mean ± SEM. (B) Contour plots show expression of IFN-γ and TNF (left) or IL-2 and IL-17A (right) of indicated experimental groups. Bar diagrams show frequencies of indicated subsets. Cytolytic activity of CD8^+^ T cells was determined on GP276-peptide loaded EL-4 target cells in a 5-hour-^51^Cr-release-assay. Symbols represent mean ± SEM. (C) Contour plots show expression of IFN-γ and TNF (left) or IL-2 and IL-17A (right) of indicated experimental groups. Bar diagrams show frequencies of indicated subsets. Statistical analysis: *p < 0.05; **p < 0.01; ***p < 0.001; ns, not significant; two-tailed unpaired Student`s *t*-test; Error bars denote mean + SEM. (A, B) Data are representative of 3 independent experiments with in total 8–9 mice per group. (C) Data are representative of 2 independent experiments with n = 2–5 mice per group.
(TIF)

S4 Fig (A) 3x10^3^ CD90.1^+^ P14 CD8 T cells from *Tbx21*^*+/+*^, *Tbx21*^*-/-*^ or *Tbx21*^*E/E*^ mice were adoptively transferred into naive CD90.2^+^ C57BL/6 recipient mice. One day later, mice were infected with 200 pfu LCMV WE. At day 8 p.i. splenocytes were analyzed by flow cytometry. (B) Viral titers in indicated organs determined by standard focus-forming assay. Error bars represent mean ± SEM. Symbols represent the values of individual mice. Dashed lines represent detection limit. (C) Contour plots depict expression of KLRG1 and CD127 of endogenous polyclonal CD8 T cells (CD8^+^ Thy1.1^-^) of indicated experimental groups. Bar diagram shows frequencies of SLEC (KLRG1^+^ CD127^-^) and MPEC (KLRG1^-^ CD127^+^) subsets. (D) Histogram shows expression of CD25 of endogenous polyclonal CD8 T cells (CD8^+^ Thy1.1^-^) and quantification of MFI of CD25 of aforementioned subset as bar diagram. (E) Bar diagrams show MFI of CD122 (top row) or CD25 (bottom row) of P14 CD8 T cells of SLEC (KLRG1^+^ CD127^-^), EEC (KLRG^-^ CD127^-^) and MPEC (KLRG1^-^ CD127^+^) subsets. Statistical analysis: *p < 0.05; **p < 0.01; ***p < 0.001; ns, not significant; two-tailed unpaired Student`s *t*-test; error bars denote mean + SEM. (A-E) Data are representative of 2 independent experiments with in total 10 mice per group.
(TIF)

S5 Fig (A) 8 days after infection with 200 pfu LCMV WE accumulation of adoptively transferred Thy1.1^+^ P14 T cells from different genotypes was analyzed by immunfluorescent staining of spleen and lymph node sections taken at x20 original magnification, Green: Thy1.1, Red: B220. (B) t-distributed stochastic neighbor embedding (t-SNE) plots based on the arcsinh-transformed expression of indicated surface markers of P14 CD8 T cells. Plots represent an overlay of *Tbx21*^*+/+*^, *Tbx21*^*-/-*^ and *Tbx21*^*E/E*^ P14 cells (20,000 cells per sample, n = 5 for each experimental group) and are stratified by indicated surface marker. Scale of each channel was transformed to values from 0 to 1.
(TIF)
