# Supplementary figures and images for: Eomes cannot replace its paralog T-bet during expansion and differentiation of CD8 effector T cells
Source: PLoS Pathog. 2020 Sep 29;16(9):e1008870. doi: 10.1371/journal.ppat.1008870 (PMC7546498; doi:10.1371/journal.ppat.1008870)

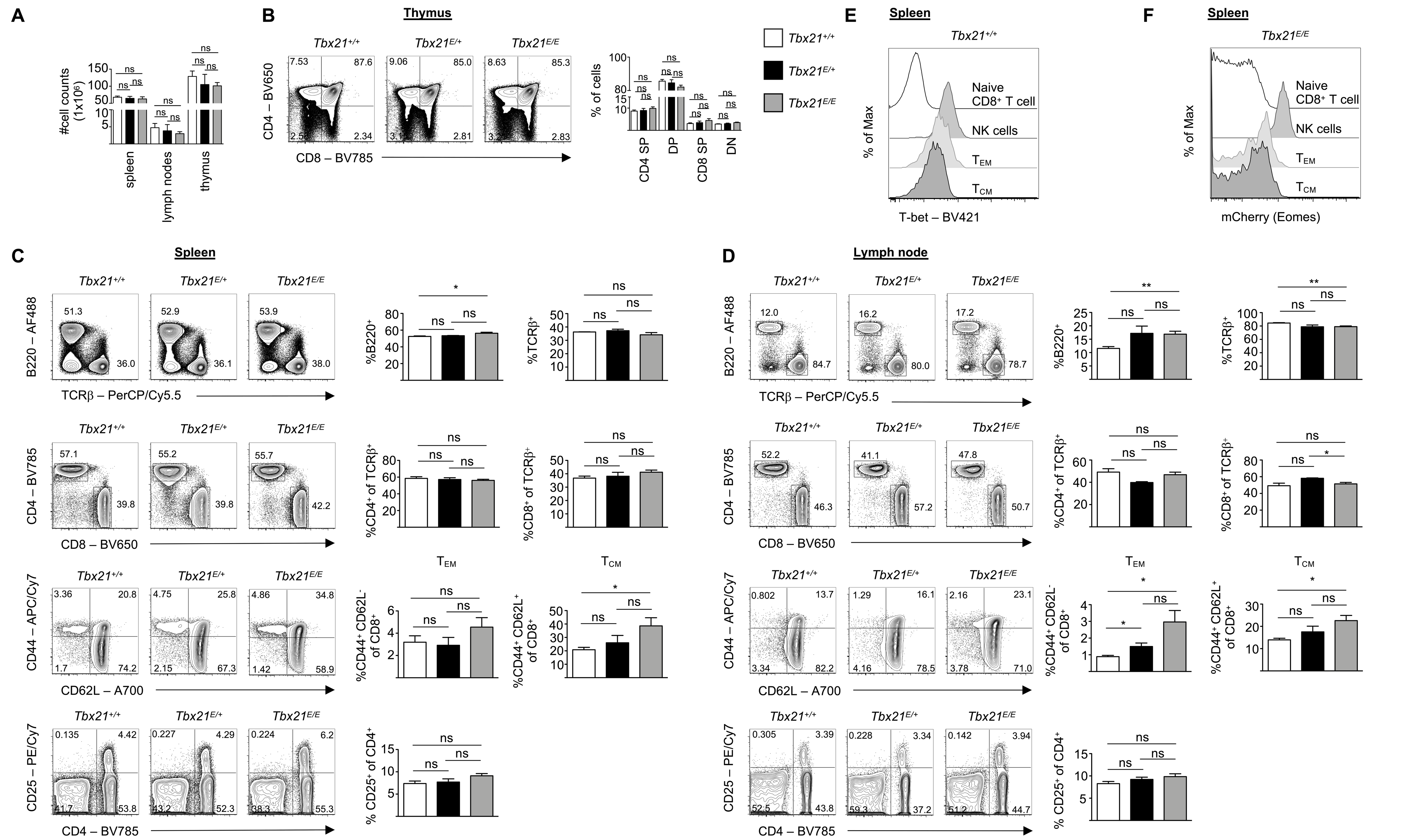

Supplement: S1 Fig — (A-D) Lymphocytes were isolated from the thymus, inguinal and axillary lymph nodes and spleen from naive 8–12 weeks old Tbx21+/+, Tbx21E/+ and Tbx21E/E mice (littermates) and analyzed by flow cytometry. (A) Bar diagram shows absolute (#) cell number of lymphocytes isolated from indicated organs and groups. (B) Contour plots show CD4 and CD8 expression of thymic cells. Bar diagram shows frequencies of CD4+ CD8- (CD4 SP) cells, CD4+ CD8+ (DP) cells, CD4- CD8+ (CD8 SP) and CD4- CD8- (DN) cells of indicated groups. (C) Contour plots and quantification of lymphocyte subsets via bar diagrams of frequencies of B cells (B220+ TCRβ-), T cells (B220- TCRβ+), CD4+ T cells (CD4+ TCRβ+), CD8+ T cells (CD8+ TCRβ+), TEM cells (CD8+ CD44+ CD62L-), TCM cells (CD8+ CD44+ CD62L+) and CD4+ CD25+ regulatory T cells (from top to bottom) isolated from the spleen of indicated groups. (D) Contour plots and quantification of lymphocyte subsets via bar diagrams of frequencies of B cells (B220+ TCRβ-), T cells (B220- TCRβ+), CD4+ T cells (CD4+ TCRβ+), CD8+ T cells (CD8+ TCRβ+), TEM cells (CD8+ CD44+ CD62L-), TCM cells (CD8+ CD44+ CD62L+) and CD4+ CD25+ regulatory T cells (from top to bottom) isolated from the inguinal and axillary lymph nodes of indicated groups. (E) Histograms show representative MFIs of T-bet of indicated lymphocyte subsets isolated from splenocytes of 8–12 weeks old Tbx21+/+ mice. (F) Histograms show representative MFIs of mCherry of indicated lymphocyte subsets isolated from splenocytes of 8–12 weeks old Tbx21E/E mice. Statistical analysis: *p < 0.05; **p < 0.01; ***p < 0.001; ns, not significant; two-tailed unpaired Student`s t-test; Error bars denote mean + SEM. (A-D) Data are representative (contour plots) or cumulative (bar diagrams) from n = 4–6 mice per group from 2 independent experiments with n = 2–4 mice per group and experiment. (TIF) [file ppat.1008870.s001.tif]

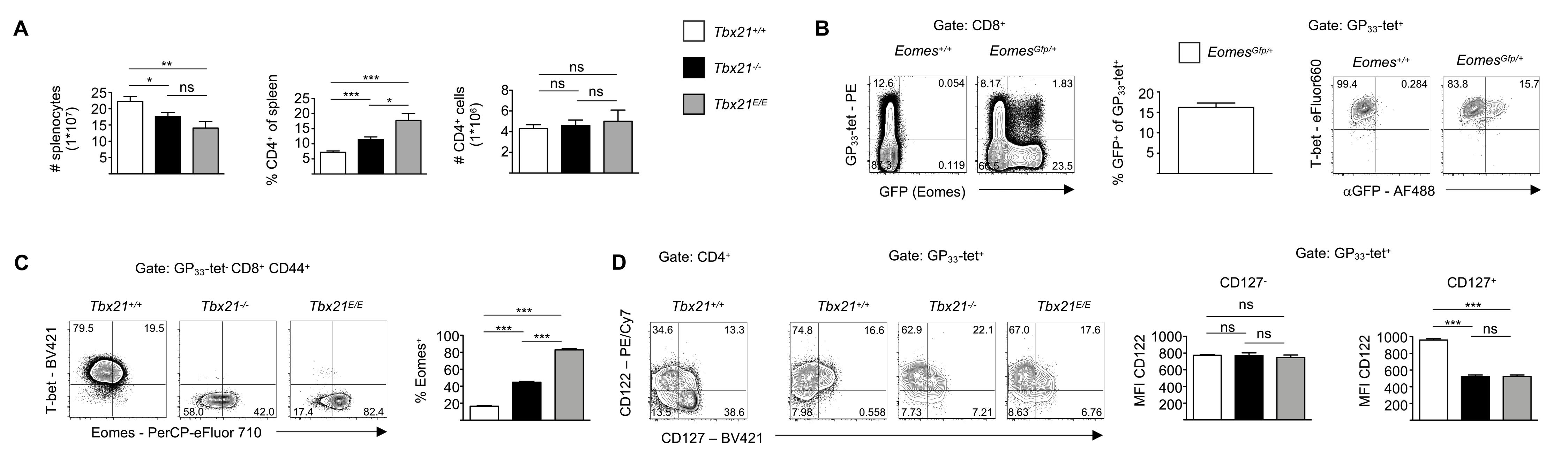

Supplement: S2 Fig — Tbx21+/+, Tbx21-/- and Tbx21E/E or Eomes+/+ and EomesGfp/+ mice were infected with 200 pfu LCMV WE. At day 8 p.i. splenocytes of indicated groups were analyzed by flow cytometry. (A) Bar diagram (left) shows absolute (#) number of splenocytes. Bar diagram (middle) shows frequency of CD4+ T cells. Right bar diagram shows absolute (#) numbers of CD4+ T cells. (B) Contour plots (left) show expression of H-2Db GP33-tetramer and GFP (Eomes). Bar diagram shows frequency of GFP+ (Eomes+) cells of GP33-tet+ cells. Contour plots (right) show expression of T-bet and Eomes after intranuclear staining of GP33-tet+ CD8+ cells. (C) Contour plots show expression of T-bet and Eomes of CD8+ CD44+ T cells as determined by flow cytometry after intranuclear staining. Quadrant gates were set according to CD44- CD4+ T cells (T-bet- Eomes- cells). Bar diagram shows percentage of Eomes+ cells. (D) Contour plots show expression of CD122 and CD127 of CD4+ T cells (left) or of GP33-tet+ CD8+ cells (right). Bar diagrams show MFI of CD122 of CD127- (left) or CD127+ (right) GP33-tet+ CD8+ cells. Statistical analysis: *p < 0.05; **p < 0.01; ***p < 0.001; ns, not significant; two-tailed unpaired Student`s t-test; Error bars denote mean + SEM. (A, C, D) Data are representative of 3 independent experiments with in total 8–9 mice per group. (B) Data are representative from 2 independent experiments with n = 3 mice per experimental group. (TIF) [file ppat.1008870.s002.tif]

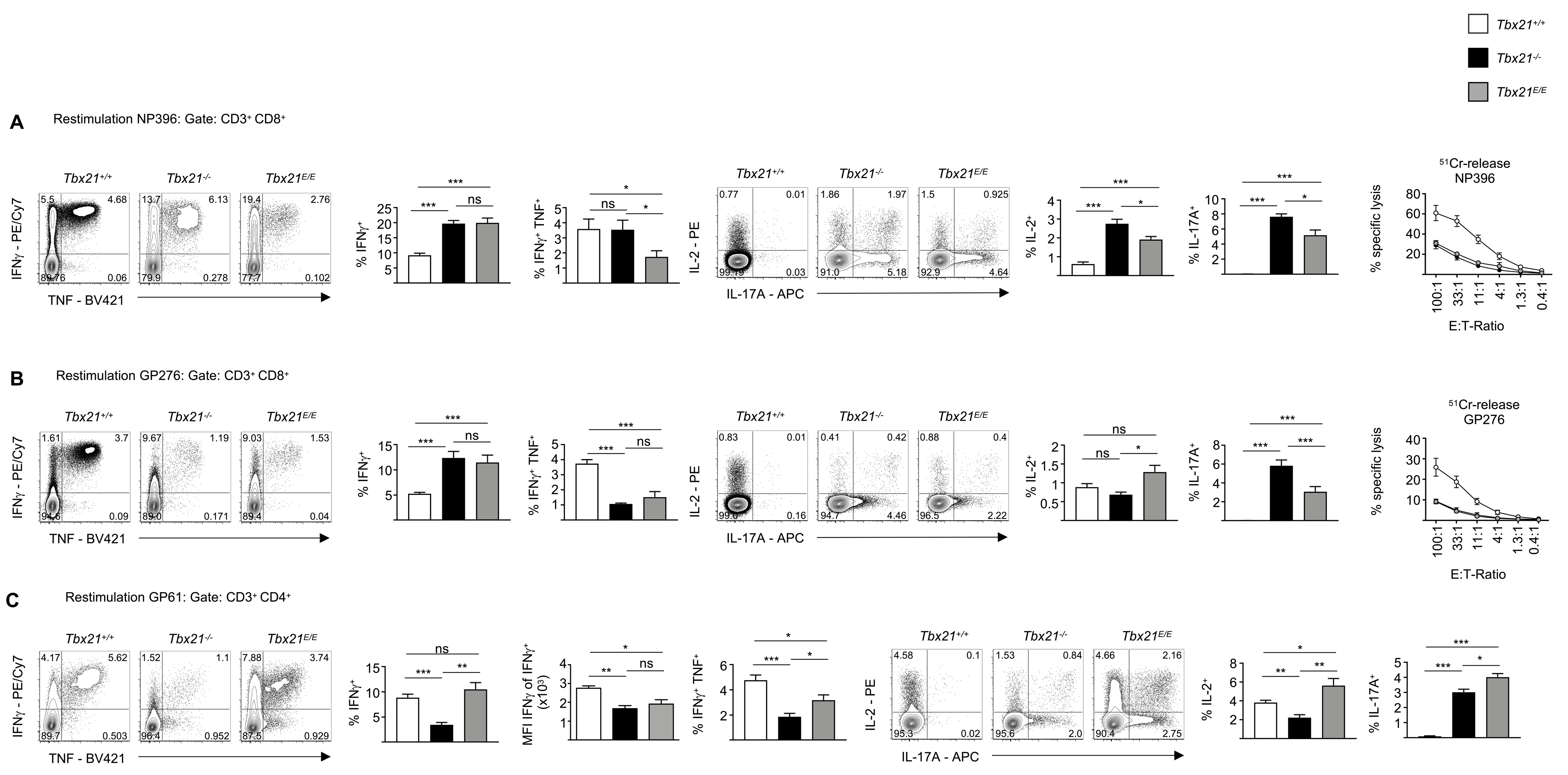

Supplement: S3 Fig — Tbx21+/+, Tbx21-/- and Tbx21E/E mice were infected with 200 pfu LCMV WE. At day 8 p.i. splenocytes were analyzed by flow cytometry. Next, splenocytes were stimulated for 5 hours with indicated peptide. Contour plots are gated on CD3+ CD8+ T cells (A, B) or CD3+ CD4+ T cells (C) and show expression of indicated cytokines after intracellular staining. Gates were set according to unstimulated cells. (A) Contour plots show expression of IFN-γ and TNF (left) or IL-2 and IL-17A (right) of indicated experimental groups. Bar diagrams show frequencies of indicated subsets. Cytolytic activity of CD8+ T cells was determined on NP396-peptide loaded EL-4 target cells in a 5-hour-51Cr-release-assay. Symbols represent mean ± SEM. (B) Contour plots show expression of IFN-γ and TNF (left) or IL-2 and IL-17A (right) of indicated experimental groups. Bar diagrams show frequencies of indicated subsets. Cytolytic activity of CD8+ T cells was determined on GP276-peptide loaded EL-4 target cells in a 5-hour-51Cr-release-assay. Symbols represent mean ± SEM. (C) Contour plots show expression of IFN-γ and TNF (left) or IL-2 and IL-17A (right) of indicated experimental groups. Bar diagrams show frequencies of indicated subsets. Statistical analysis: *p < 0.05; **p < 0.01; ***p < 0.001; ns, not significant; two-tailed unpaired Student`s t-test; Error bars denote mean + SEM. (A, B) Data are representative of 3 independent experiments with in total 8–9 mice per group. (C) Data are representative of 2 independent experiments with n = 2–5 mice per group. (TIF) [file ppat.1008870.s003.tif]

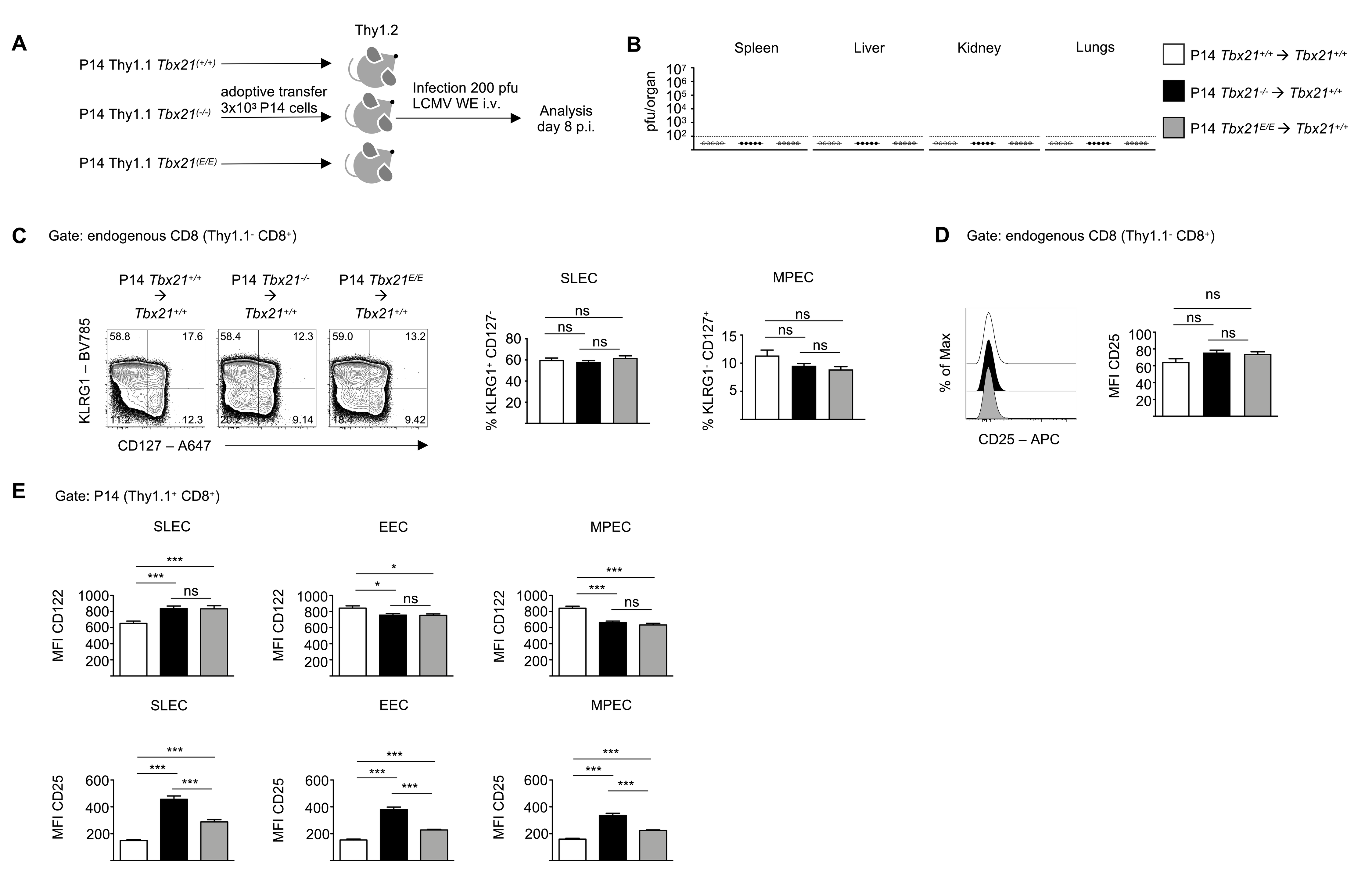

Supplement: S4 Fig — (A) 3x10^3^ CD90.1+ P14 CD8 T cells from Tbx21+/+, Tbx21-/- or Tbx21E/E mice were adoptively transferred into naive CD90.2+ C57BL/6 recipient mice. One day later, mice were infected with 200 pfu LCMV WE. At day 8 p.i. splenocytes were analyzed by flow cytometry. (B) Viral titers in indicated organs determined by standard focus-forming assay. Error bars represent mean ± SEM. Symbols represent the values of individual mice. Dashed lines represent detection limit. (C) Contour plots depict expression of KLRG1 and CD127 of endogenous polyclonal CD8 T cells (CD8+ Thy1.1-) of indicated experimental groups. Bar diagram shows frequencies of SLEC (KLRG1+ CD127-) and MPEC (KLRG1- CD127+) subsets. (D) Histogram shows expression of CD25 of endogenous polyclonal CD8 T cells (CD8+ Thy1.1-) and quantification of MFI of CD25 of aforementioned subset as bar diagram. (E) Bar diagrams show MFI of CD122 (top row) or CD25 (bottom row) of P14 CD8 T cells of SLEC (KLRG1+ CD127-), EEC (KLRG- CD127-) and MPEC (KLRG1- CD127+) subsets. Statistical analysis: *p < 0.05; **p < 0.01; ***p < 0.001; ns, not significant; two-tailed unpaired Student`s t-test; error bars denote mean + SEM. (A-E) Data are representative of 2 independent experiments with in total 10 mice per group. (TIF) [file ppat.1008870.s004.tif]

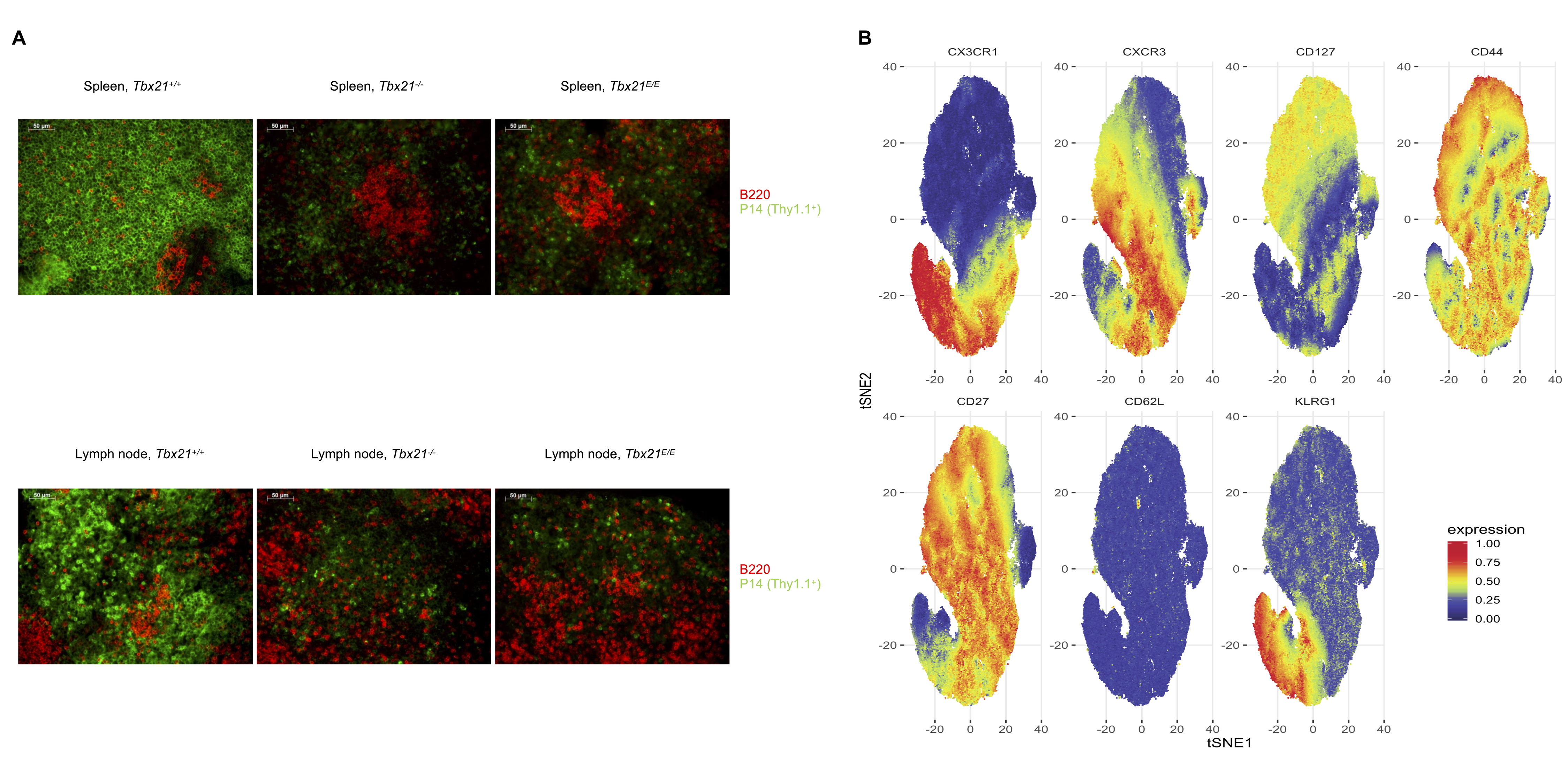

Supplement: S5 Fig — (A) 8 days after infection with 200 pfu LCMV WE accumulation of adoptively transferred Thy1.1+ P14 T cells from different genotypes was analyzed by immunfluorescent staining of spleen and lymph node sections taken at x20 original magnification, Green: Thy1.1, Red: B220. (B) t-distributed stochastic neighbor embedding (t-SNE) plots based on the arcsinh-transformed expression of indicated surface markers of P14 CD8 T cells. Plots represent an overlay of Tbx21+/+, Tbx21-/- and Tbx21E/E P14 cells (20,000 cells per sample, n = 5 for each experimental group) and are stratified by indicated surface marker. Scale of each channel was transformed to values from 0 to 1. (TIF) [file ppat.1008870.s005.tif]
